# Supplementary material for: Optimization of 3-Cyano-7-cyclopropylamino-pyrazolo[1,5-a]pyrimidines Toward the Development of an In Vivo Chemical Probe for CSNK2A
Source: bioRxiv. 2023 May 18:2023.05.15.540828. Preprint. [Version 1] doi: 10.1101/2023.05.15.540828 (PMC10245575; doi:10.1101/2023.05.15.540828)
Supplement: Supplementary file 1 [file media-1.pdf]

SUPPORTING INFORMATION

Optimization of 3-Cyano-7-cyclopropylamino-pyrazolo[1,5-a]pyrimidines Toward the Development of an In Vivo Chemical Probe for CSNK2A

Xuan Yang,[a,b] Han Wee Ong,[a,b] Rebekah J. Dickmander,[b.c.d.e] Jeffery L. Smith,[a] Jason W. Brown,[f] William Tao,[f] Edcon Chang,[f] Nathaniel J. Moorman,[b,c,d] Alison D. Axtman,[a,b] and Timothy M. Willson[a,b,*]

[a] *Structural Genomics Consortium, UNC Eshelman School of Pharmacy, University of North Carolina at Chapel Hill, Chapel Hill, NC 27599, United States*

[b] *Rapidly Emerging Antiviral Drug Development Initiative (READDI), Chapel Hill, NC 27599, United States*

[c] *Department of Microbiology & Immunology, University of North Carolina at Chapel Hill, Chapel Hill, NC 27599, United States*

[d] *Lineberger Comprehensive Cancer Center, University of North Carolina at Chapel Hill, Chapel Hill, NC 27599, United States*

[e] *Department of Chemistry, University of North Carolina at Chapel Hill, Chapel Hill, NC 27599, United States*

[f] *Takeda San Diego, San Diego, CA 92121, United States*

***** E-mail: tim.willson@unc.edu

Table of Contents

S2 **Figure S1.** SMARTCyp analysis of SGC-CK2-1 (**1a**)

S3 **Figure S2.** 1-ABT inhibits the metabolism of **2h** in mouse hepatocytes

S4 **Table S1.** Dose ranging study of EA tolerability in mice

S5–33 NMR and HPLC Spectra for **1h–i**, **2b–g, 2i–n**

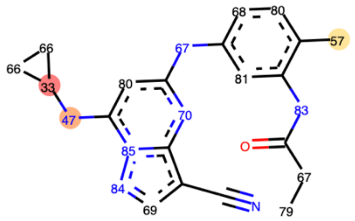

3A4 Ranking	Atom	3A4 Score	Energy	2DSASA	Span2end	Relative Span	Similarity
1	C.21	33.0	41.1	17.1	1	0.9	0.3
2	N.20	46.8	54.1	13.5	2	0.8	0.3
3	C.28	57.3	66.4	58.7	2	0.8	0.7
4	C.22	65.9	75.9	50.1	0	1.0	0.3
5	N.9	67.2	72.0	12.3	6	0.5	0.7
6	C.3	67.3	75.9	30.6	1	0.9	0.7
7	C.25	68.1	74.1	26.4	5	0.6	0.7
8	C.16	69.4	78.1	33.4	1	0.9	0.3
9	N.11	69.6	75.6	11.0	4	0.7	0.3
10	C.4	79.0	89.6	64.4	0	1.0	0.7
11	C.26	79.7	86.3	27.5	4	0.7	0.7
12	C.24	80.0	86.3	18.6	4	0.7	0.3
13	C.7	80.6	86.3	19.9	5	0.6	0.3
14	N.5	83.0	89.6	10.8	3	0.8	0.3
15	N.17	83.8	92.1	22.1	1	0.9	0.3
16	N.18	85.2	92.1	3.3	2	0.8	0.3

Figure S1. SMARTCyp analysis of SGC-CK2-1 (**1a**). Output from the SMARTCyp server at https://smartcyp.sund.ku.dk/mol_to_som. The predicted hotspots of cytochrome CYP3A4 metabolism are ranked for each heavy atom in the molecule. The top ranked atoms are located on the cycloproylamine of the PZP and the 4’-methyl group of the aniline.

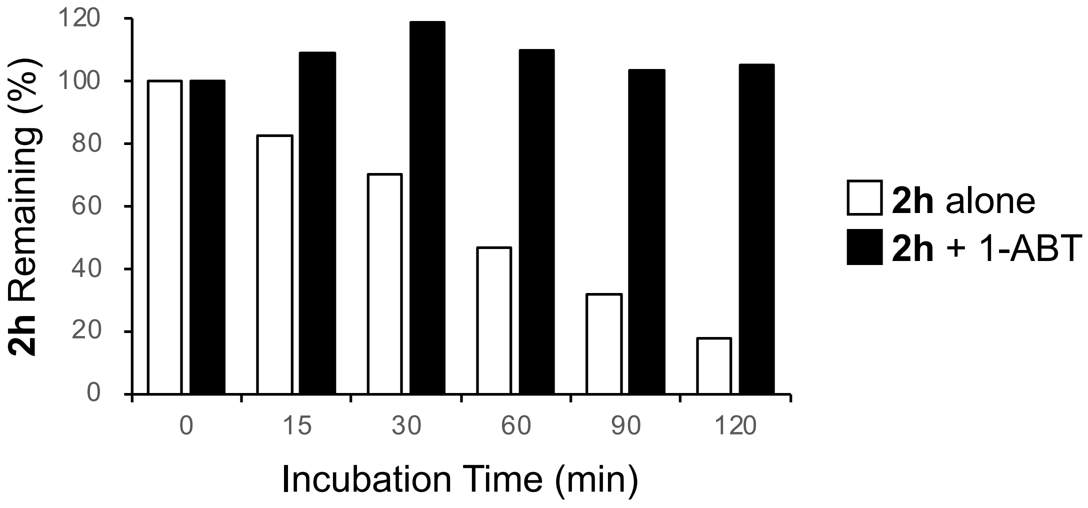

Figure S2. 1-ABT inhibits the metabolism of **2h** in mouse hepatocytes. **2h** (1 μM) was incubated with mouse hepatocytes in the presence or absence of 1-ABT (1 mM). The percentage of **2h** remaining was measured by LC/MS/MS at 0.25, 0.5, 1.0, 1.5, and 2.0h.

Table S1. Dose ranging study of EA tolerability in mice.

Dose (mg/kg)	Route	Volume (mL/kg)[a]	Formul-ation[b]	Adverse effects after 1st dose[c]	Adverse effects after 2nd dose[c]
300	i.p.	10	A	Tachycardia, hypothermia, death @ 15 min	–
100	i.p.	10	A	Decreased activity, hypothermia, death @ 40 min	–
30	i.p.	10	A	Decreased activity	Decreased activity, death @ 60 min
10	i.p.	10	A	Decreased activity	Decreased activity, hypothermia, death @ 70 min
3	i.p.	10	A	Decreased activity	Decreased activity, hypothermia
10	i.p.	1	B	None	Decreased activity, hypothermia[d]
10	p.o.	1	B	None	Decreased activity, hypothermia[d]

Mice were dosed at 6h intervals with EA and observations made of clinical symptoms. A single mouse was used for each experiment. [a]Injection volume. [b]Formulation A: NMP, PEG400, Water (v/v/v, 10:60:30), Formulation B: DMSO, PEG300, Tween80, 20% HP-β-CD in water (v/v/v/v, 10:40:5:45). [c]Clinical observations with time of death after dosing noted. [d]Symptoms resolved after 30 min. –, no second dose.

NMR and HPLC spectra of 1h:

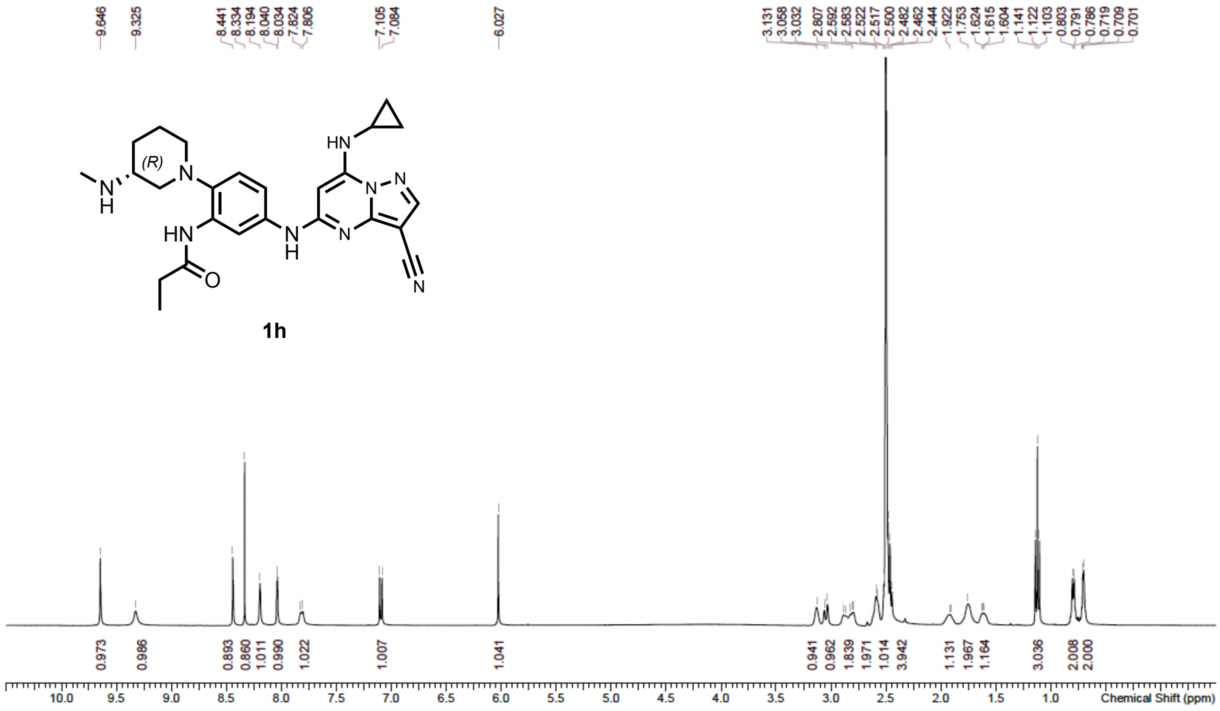

1h

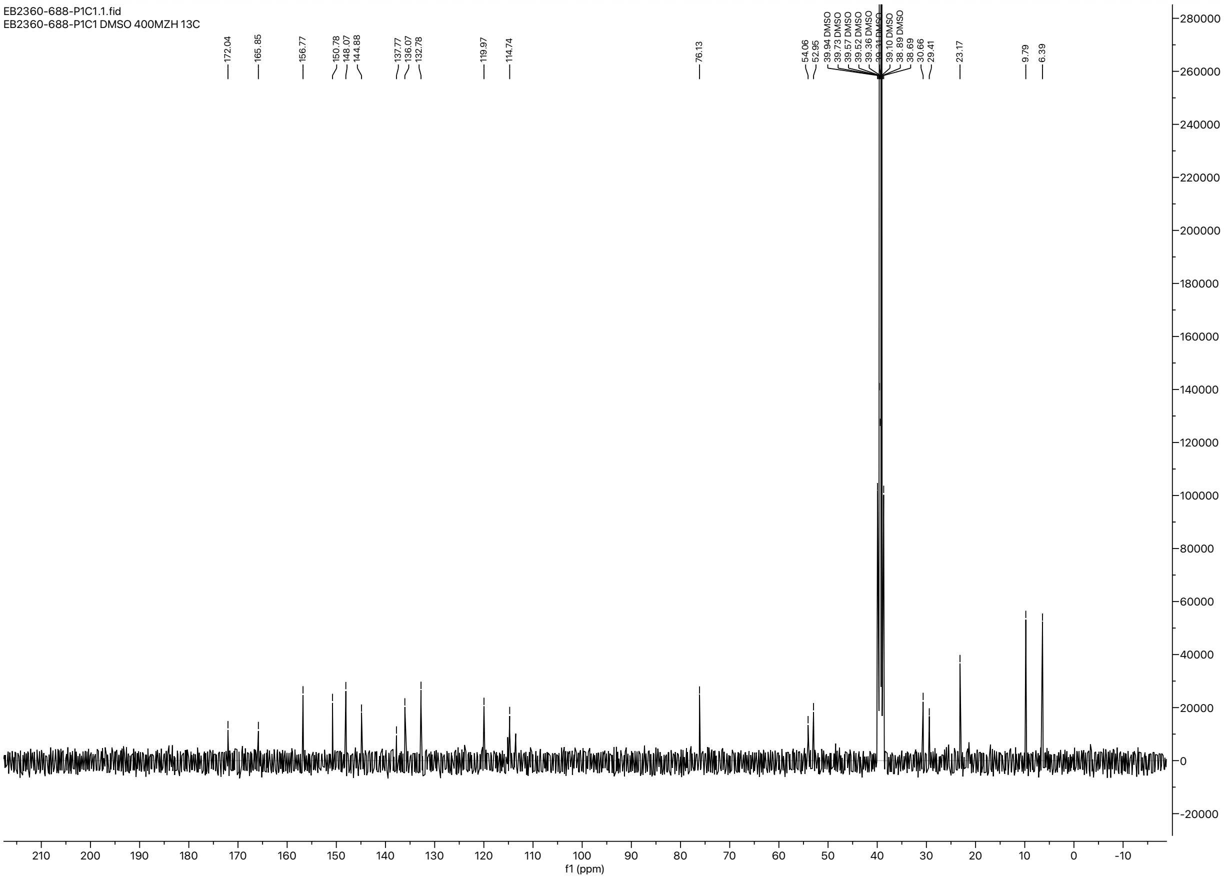

Chromatogram
WX-TKDU-F_009 D:\DATA\2021\2109\210915\EB2360-688-P1H1.lcd

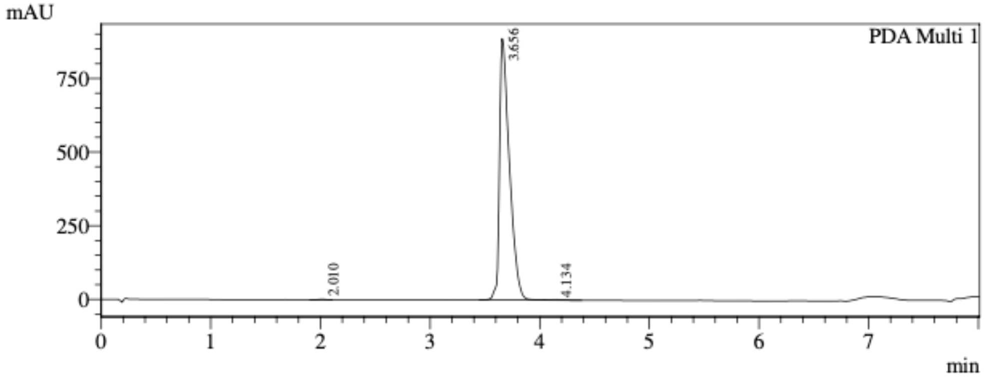

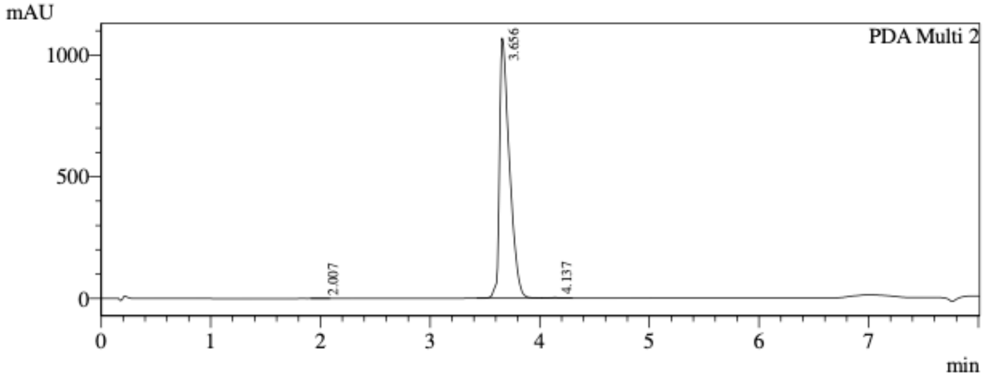

1 PDA Multi 1 / 220nm 4nm
2 PDA Multi 2 / 254nm 4nm

Integration Result

PeakTable

Ch1 220nm 4nm

Peak#	Ret. Time	Height	Height %	Area	Area %
1	2.010	1586	0.178	8125	0.145
2	3.656	886751	99.581	5578720	99.572
3	4.134	2145	0.241	15832	0.283
Total		890482	100.000	5602677	100.000

PeakTable

Ch2 254nm 4nm

Peak#	Ret. Time	Height	Height %	Area	Area %
1	2.007	596	0.056	3088	0.046
2	3.656	1068344	99.725	6733801	99.686
3	4.137	2348	0.219	18112	0.268
Total		1071288	100.000	6755001	100.000

PeakTable

Ch3

NMR and HPLC spectra of 1i:

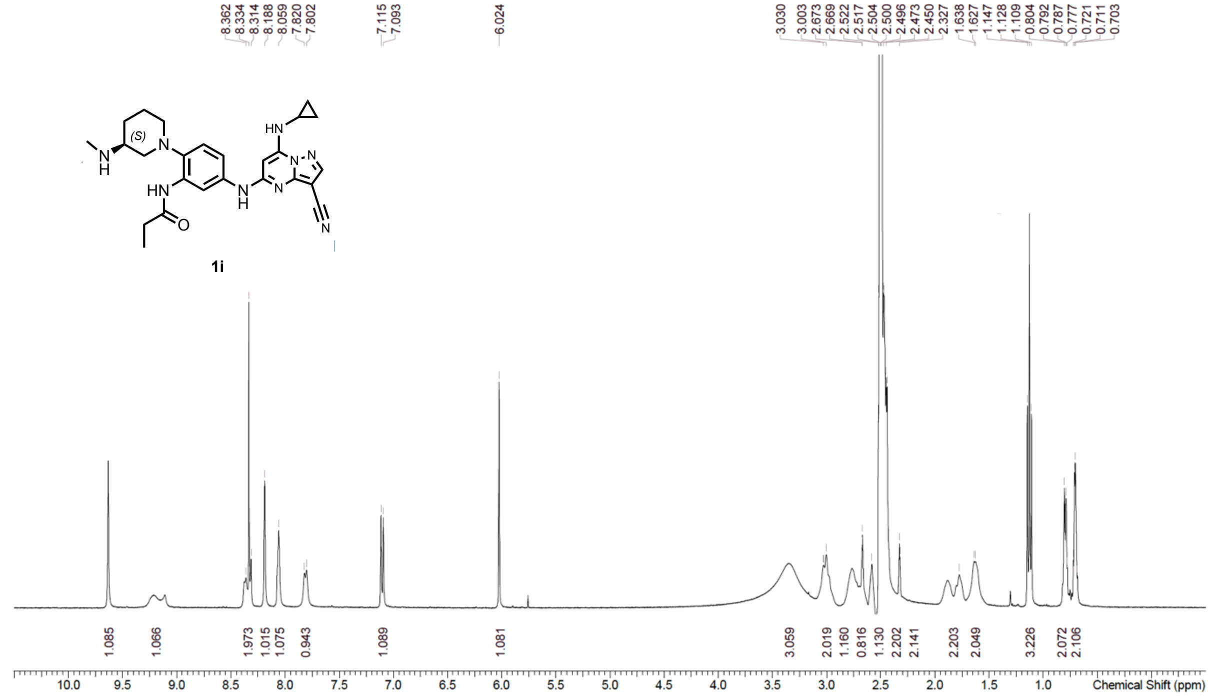

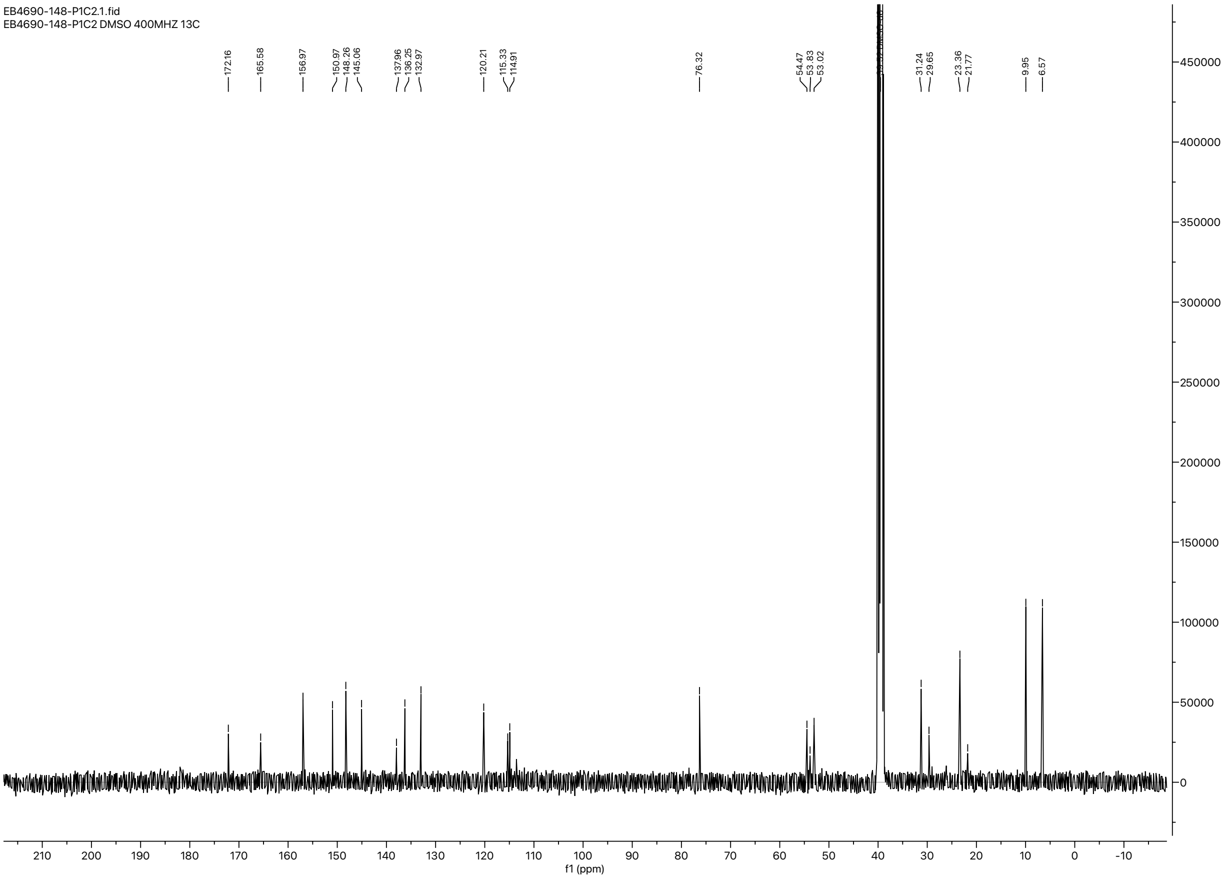

<Chromatogram>

Chromatogram
WX-TKDU-F_010 D:\DATA\2021\2109\210916\EB4690-148-P1H1.lcd

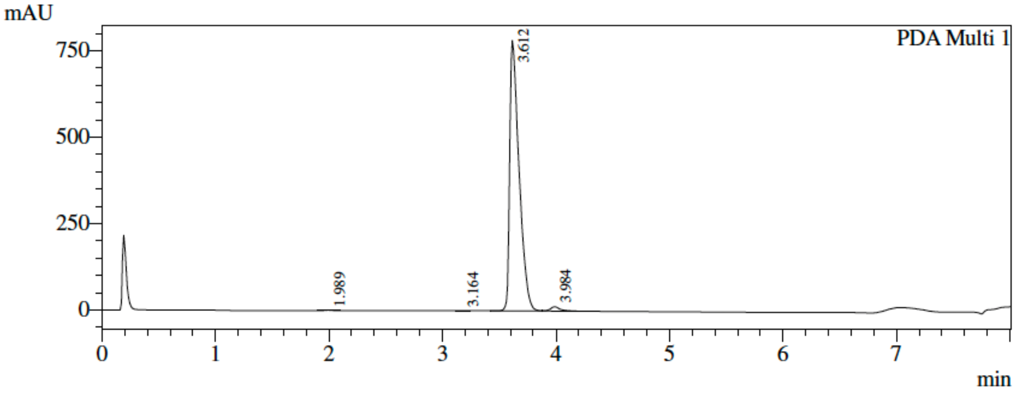

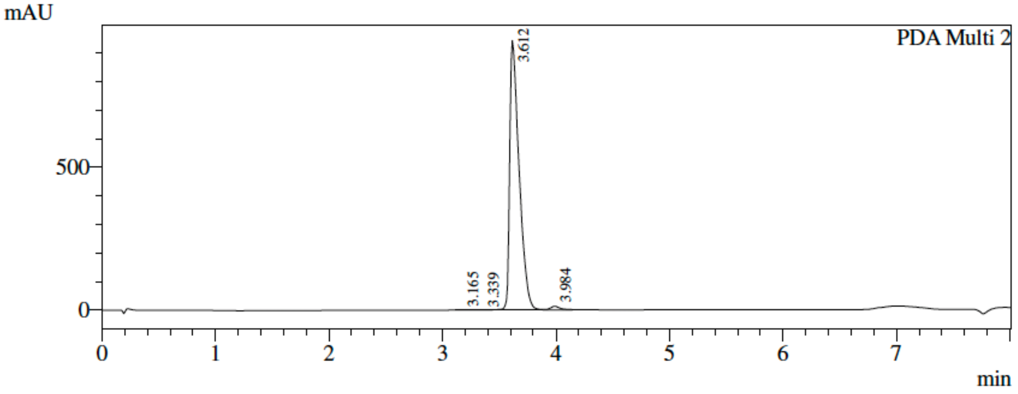

1 PDA Multi 1 / 220nm 4nm
2 PDA Multi 2 / 254nm 4nm

Integration Result

PeakTable

Ch1 220nm 4nm

Peak#	Ret. Time	Height	Height %	Area	Area %
1	1.989	1411	0.177	5667	0.123
2	3.164	842	0.106	2792	0.061
3	3.612	781856	98.124	4505087	98.071
4	3.984	12697	1.593	80171	1.745
Total		796806	100.000	4593717	100.000

PeakTable

Ch2 254nm 4nm

Peak#	Ret. Time	Height	Height %	Area	Area %
1	3.165	615	0.064	2024	0.037
2	3.339	524	0.055	4383	0.079
3	3.612	942218	98.585	5445281	98.407
4	3.984	12384	1.296	81740	1.477
Total		955740	100.000	5533428	100.000

PeakTable

Ch3

NMR and HPLC spectra of 2a:

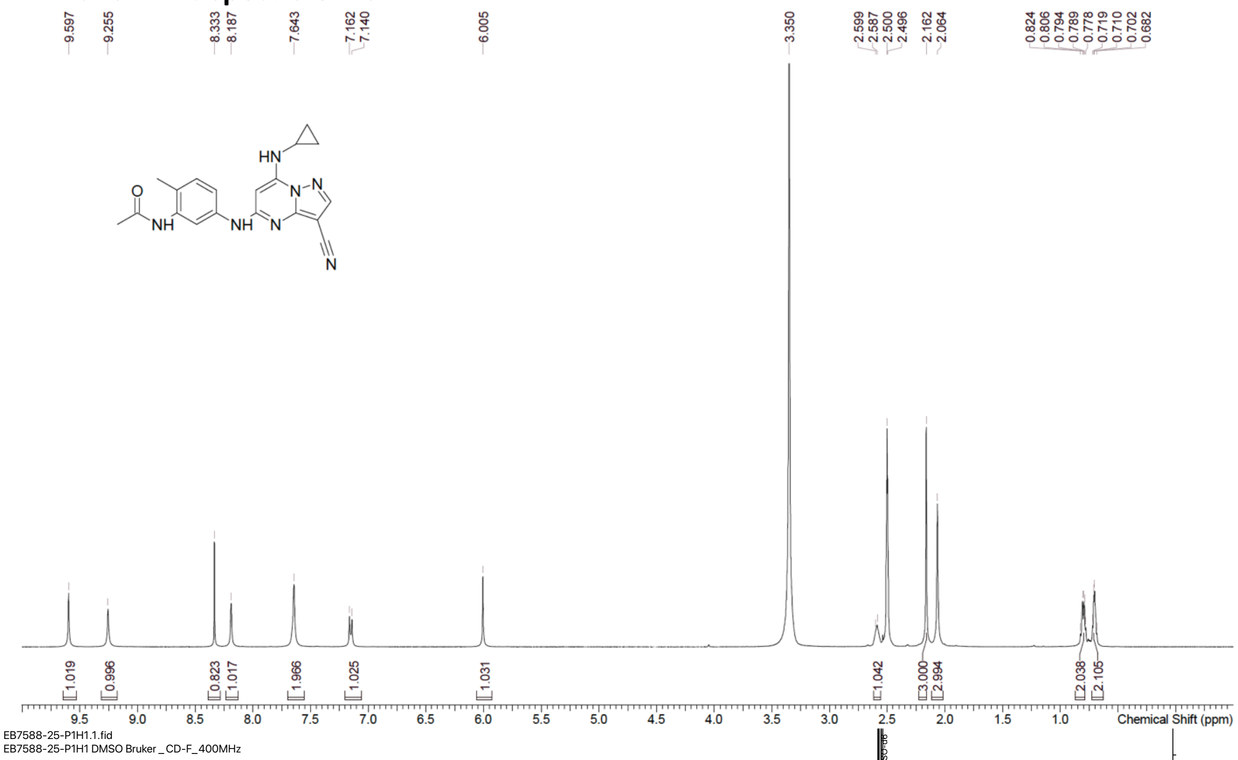

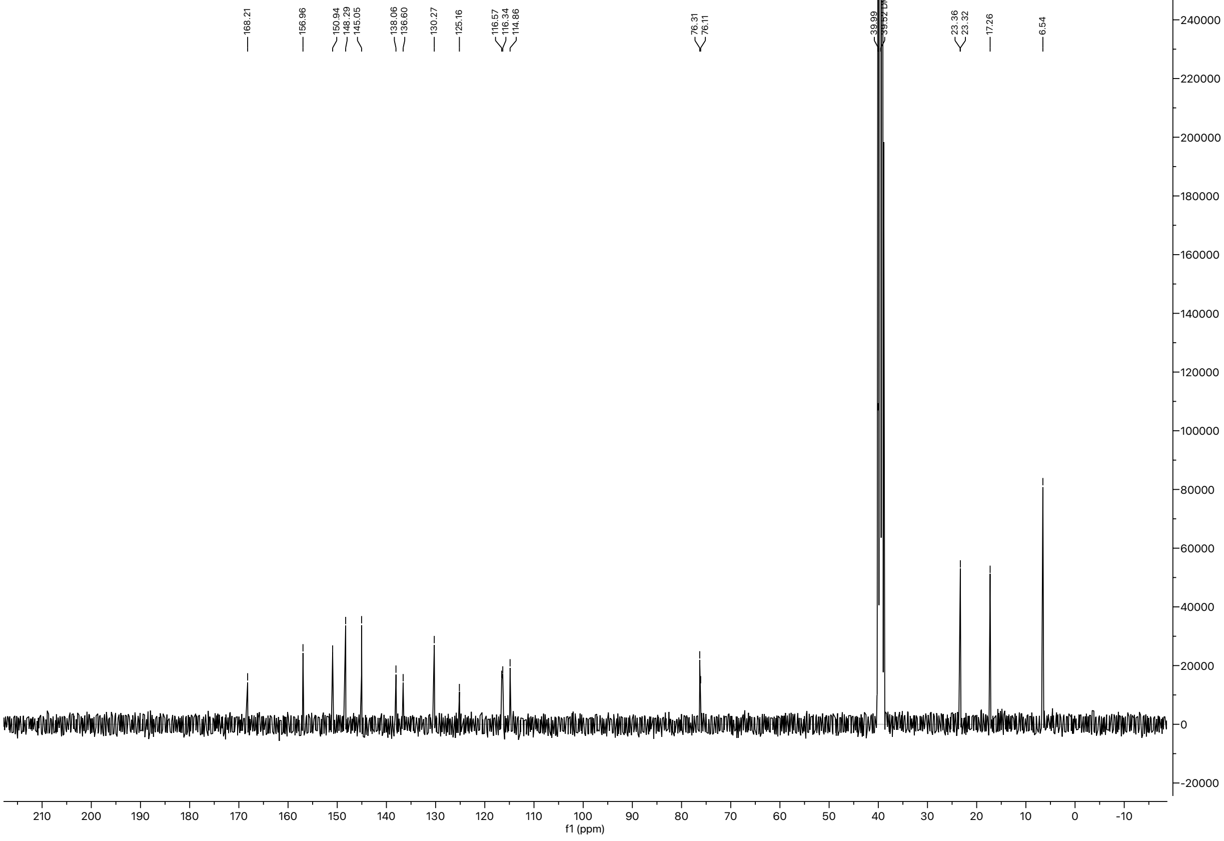

Chromatogram

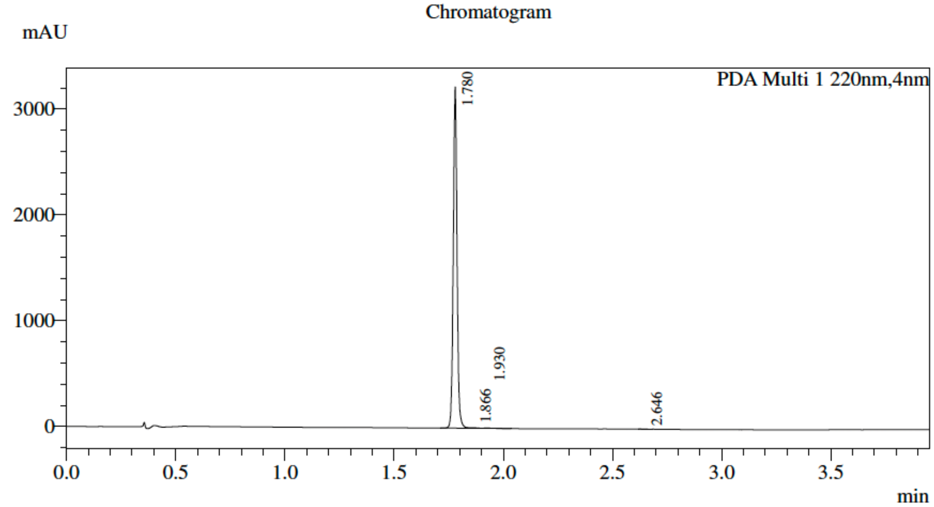

==

Integration Result

==

PDA Ch1 220nm

Peak#	Ret. Time	USP Width	Height	Height%	Area	Area%
1	1.780	0.032	3226270	99.670	3879046	99.446
2	1.866	0.080	5152	0.159	9754	0.250
3	1.930	0.066	3910	0.121	9447	0.242
4	2.646	0.039	1610	0.050	2390	0.061
Total			3236942	100.000	3900637	100.000

Chromatogram

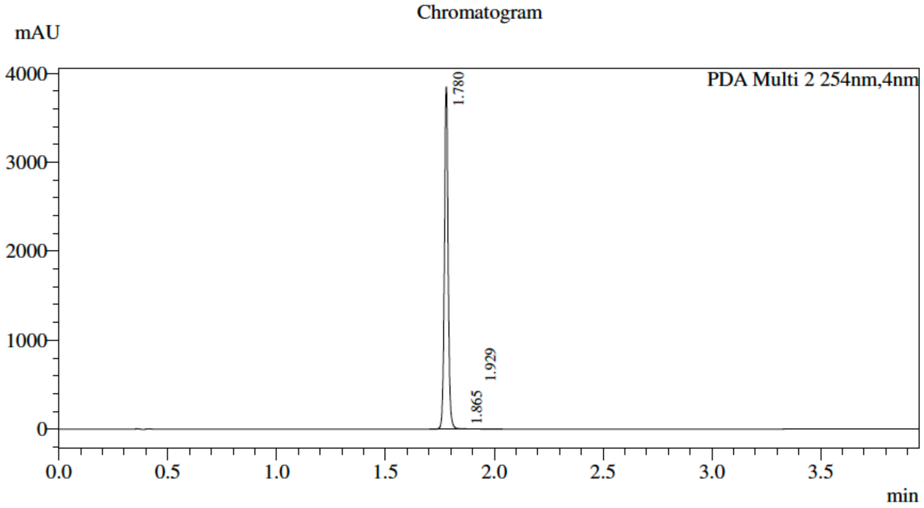

==

Integration Result

==

PDA Ch2 254nm

Peak#	Ret. Time	USP Width	Height	Height%	Area	Area%
1	1.780	0.028	3846935	99.780	4569098	99.570
2	1.865	0.101	5193	0.135	10743	0.234
3	1.929	0.097	3295	0.085	8985	0.196
Total			3855422	100.000	4588826	100.000

NMR and HPLC spectra of 2b:

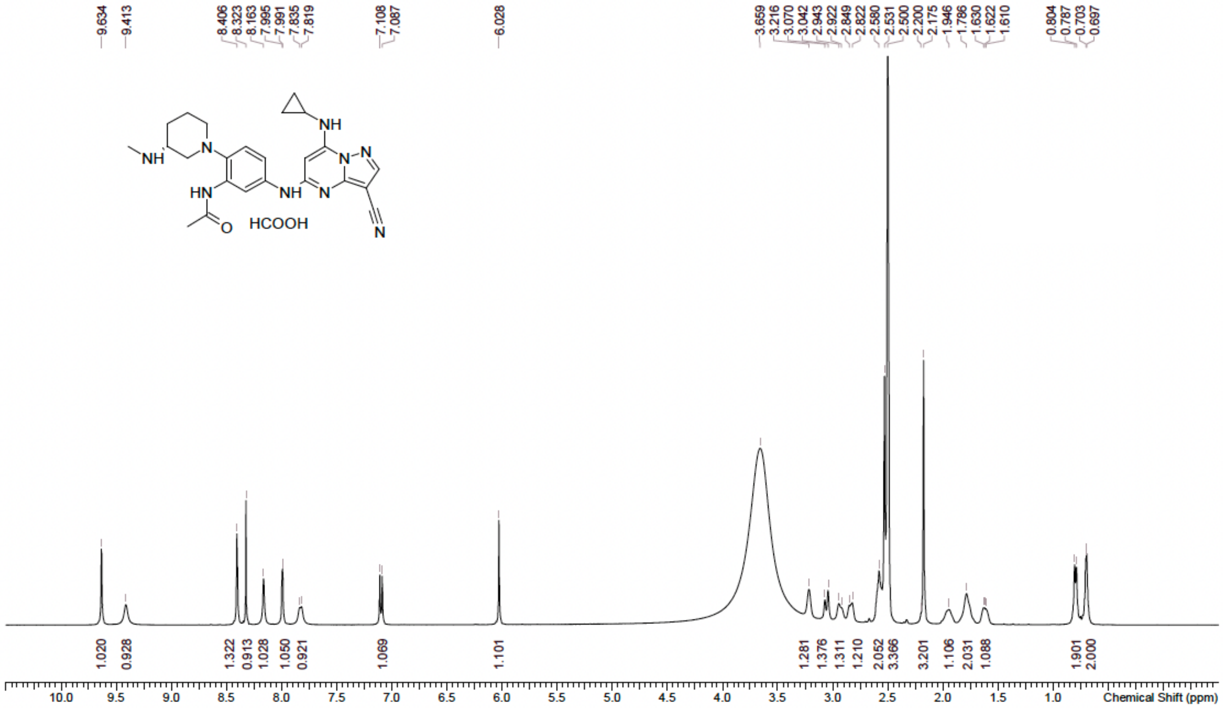

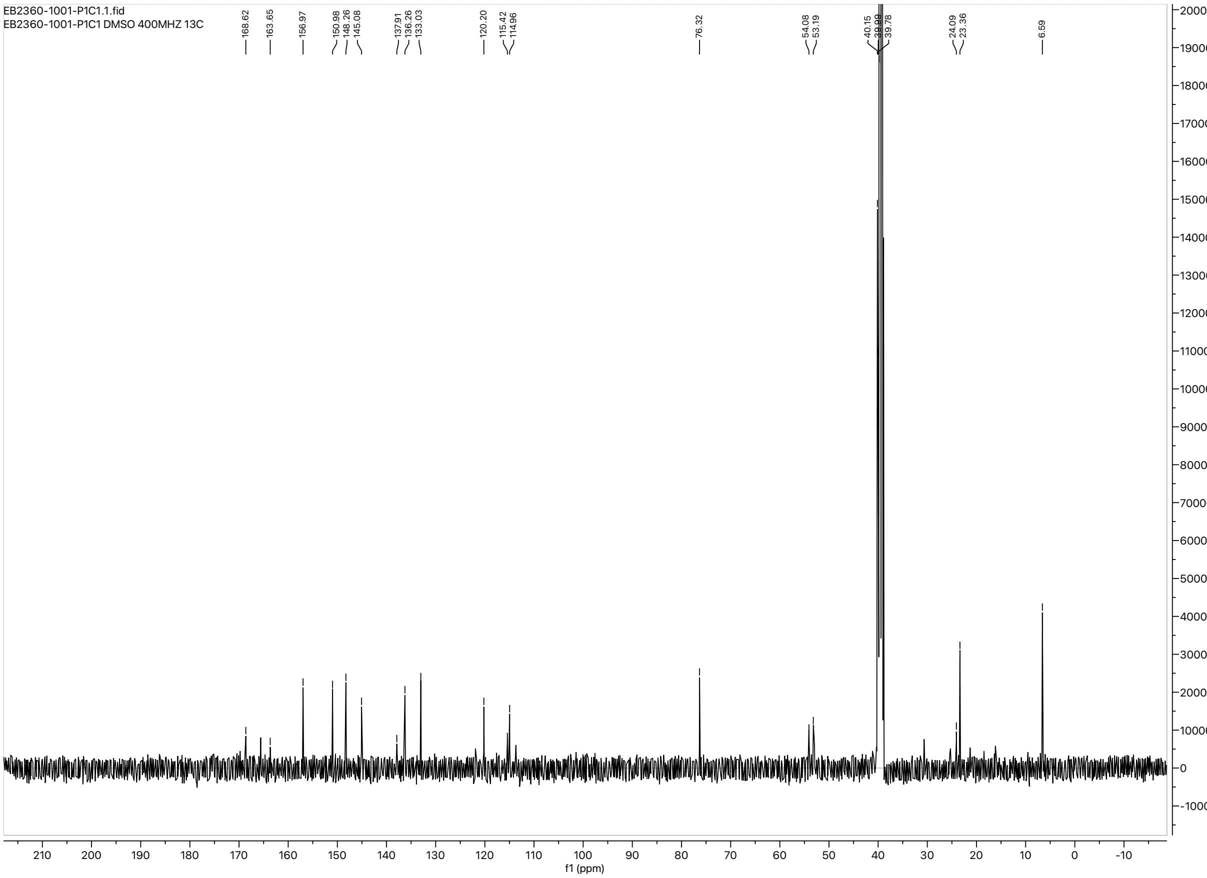

<Chromatogram>

Chromatogram
WX-TKDU-Y_025 D:\DATA\2022\2205\220504\EB2360-1001-P1H1.lcd

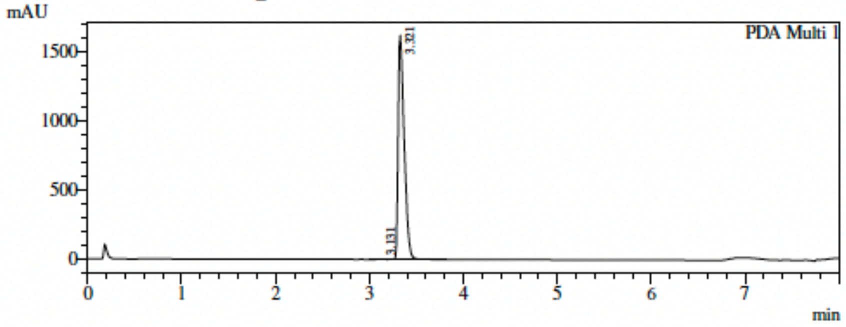

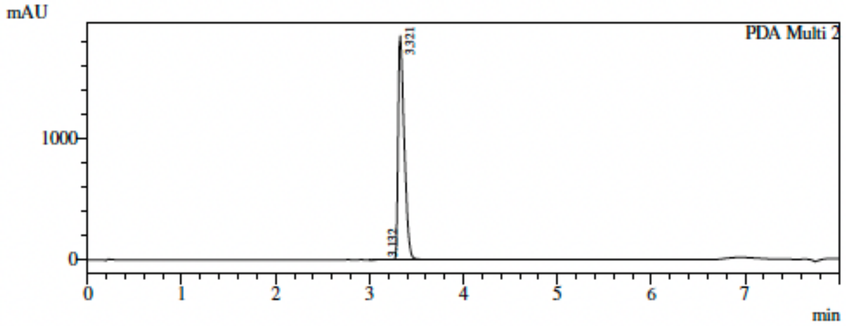

1 PDA Multi 1 / 220nm 4nm
2 PDA Multi 2 / 254nm 4nm

Integration Result

PeakTable

Ch1 220nm 4nm

Peak#	Ret. Time	Height	Height %	Area	Area %
1	3.131	1033	0.064	6654	0.091
2	3.321	1622252	99.936	7297195	99.909
Total		1623285	100.000	7303848	100.000

PeakTable

Ch2 254nm 4nm

Peak#	Ret. Time	Height	Height %	Area	Area %
1	3.132	1380	0.074	8612	0.103
2	3.321	1854411	99.926	8393331	99.897
Total		1855790	100.000	8401943	100.000

PeakTable

Ch3

NMR and HPLC spectra of 2c:

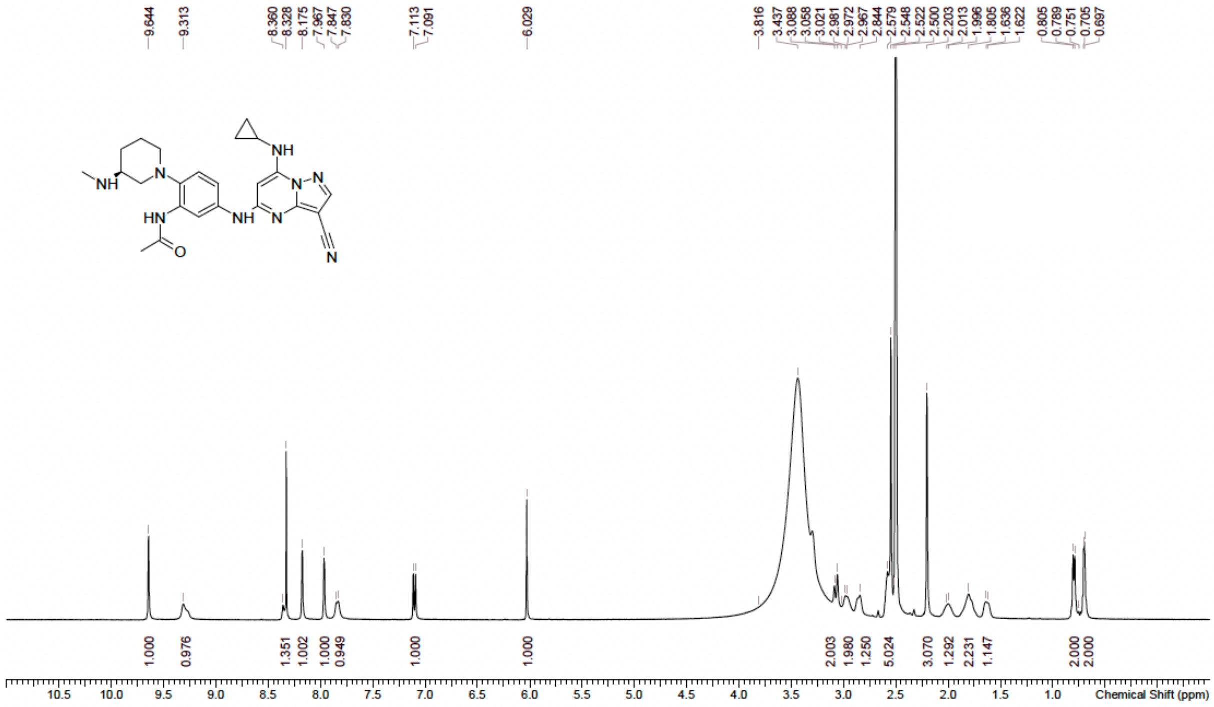

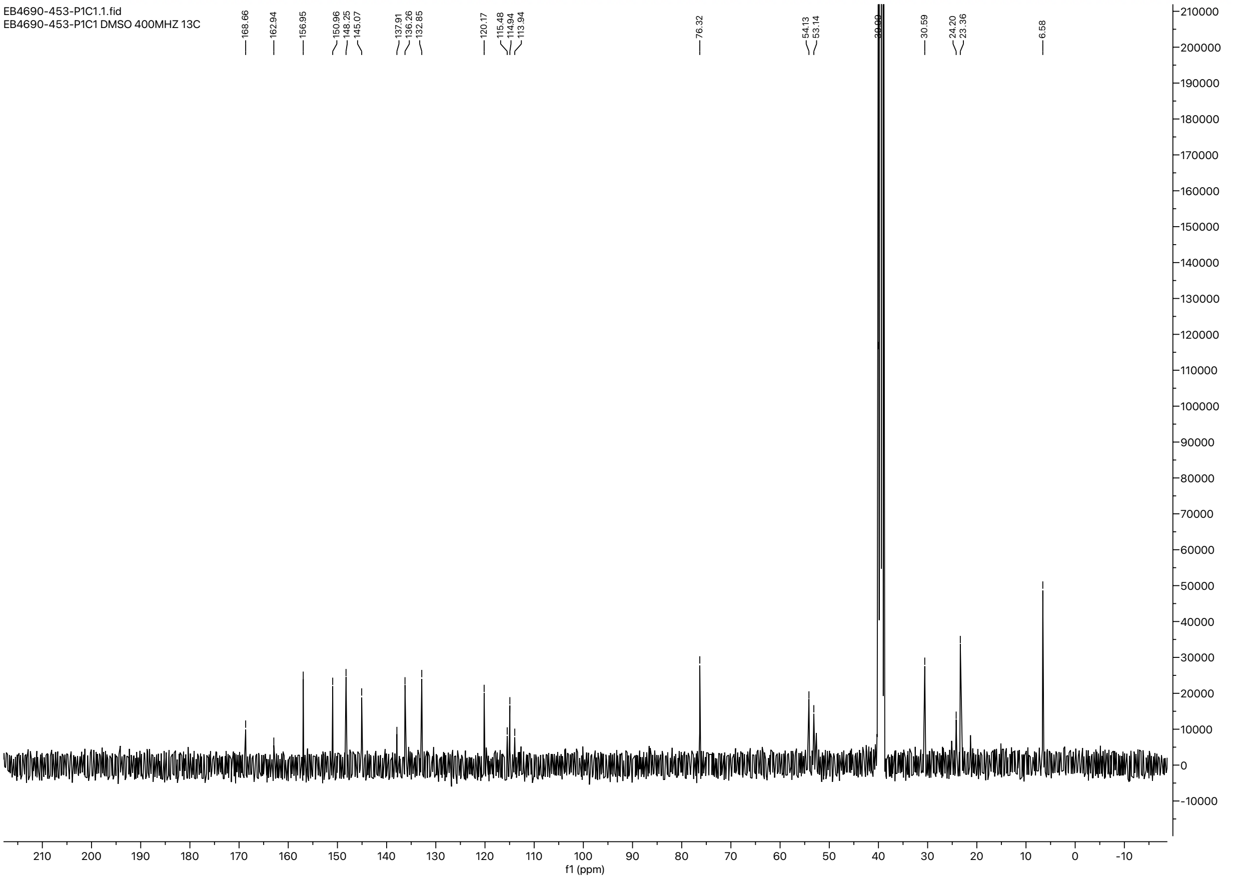

<Chromatogram>

Chromatogram
WX-TKDU-Y_026 D:\DATA\2022\2205\220505\EB4690-453-P1H1.lcd

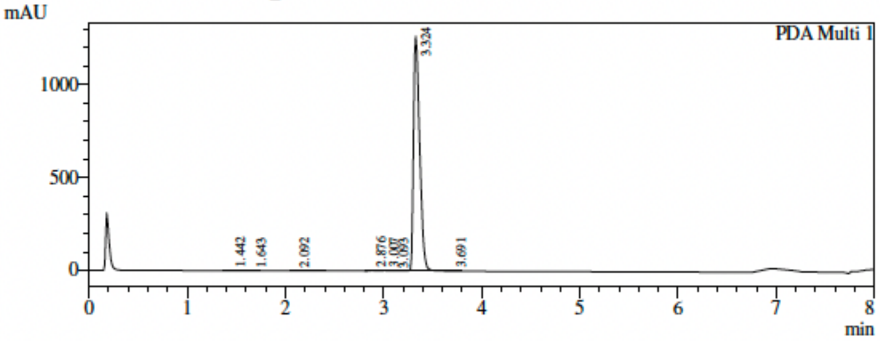

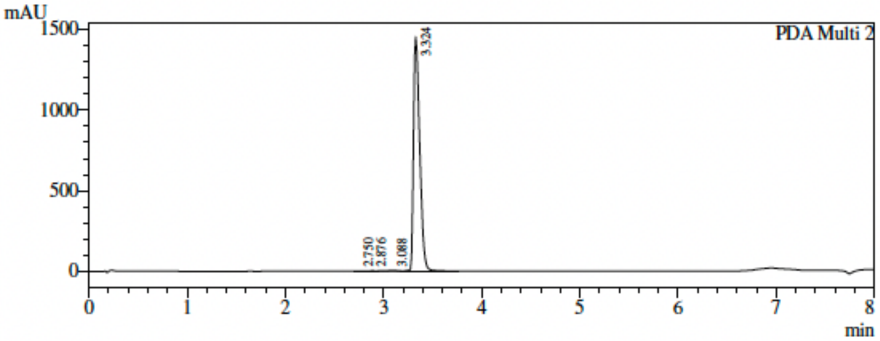

1 PDA Multi 1 / 220nm 4nm
2 PDA Multi 2 / 254nm 4nm

Integration Result

Peak#	Ret. Time	Height	Height %	Area	Area %
1	1.442	669	0.052	3821	0.068
2	1.643	904	0.071	3964	0.071
3	2.092	936	0.073	4694	0.084
4	2.876	2818	0.221	8102	0.145
5	3.007	2896	0.227	11392	0.204
6	3.093	2306	0.181	9965	0.178
7	3.324	1262348	99.123	5553022	99.206
8	3.691	642	0.050	2505	0.045
Total		1273520	100.000	5597466	100.000

PeakTable

Ch2 254nm 4nm

Peak#	Ret. Time	Height	Height %	Area	Area %
1	2.750	704	0.048	1976	0.031
2	2.876	2048	0.141	5918	0.092
3	3.088	2344	0.161	17110	0.266
4	3.324	1452495	99.650	6407337	99.611
Total		1457591	100.000	6432342	100.000

PeakTable

Ch3

NMR and HPLC spectra of 2d:

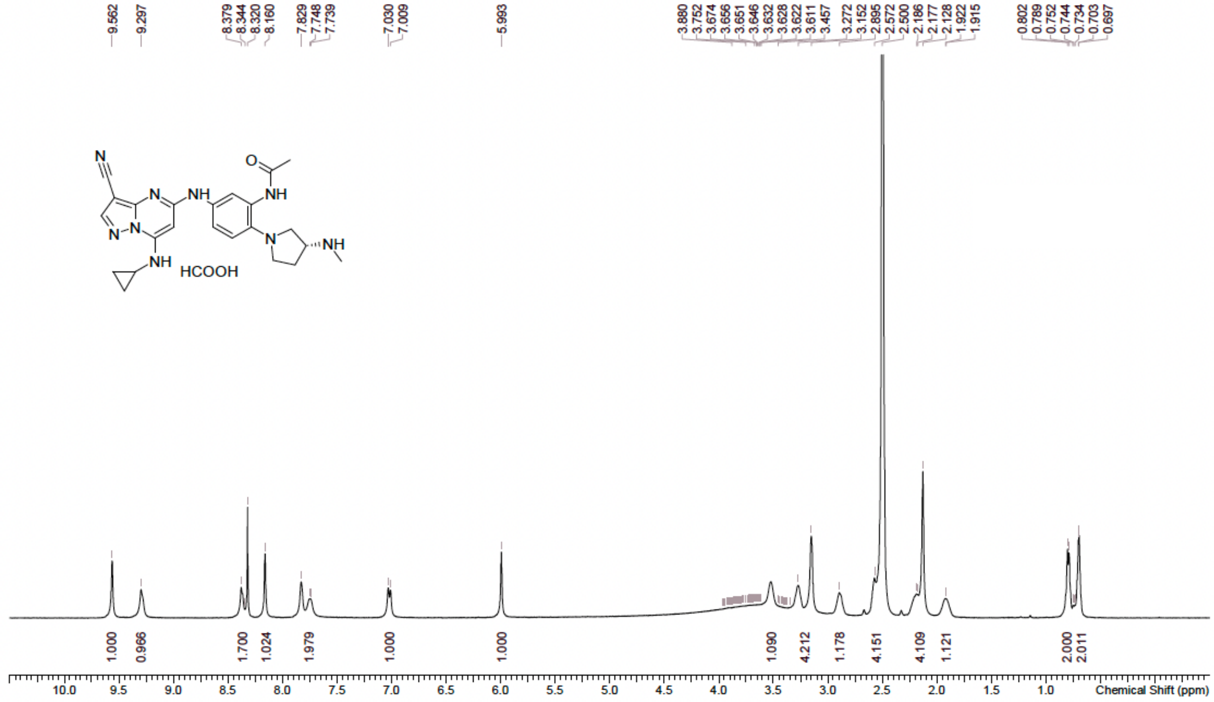

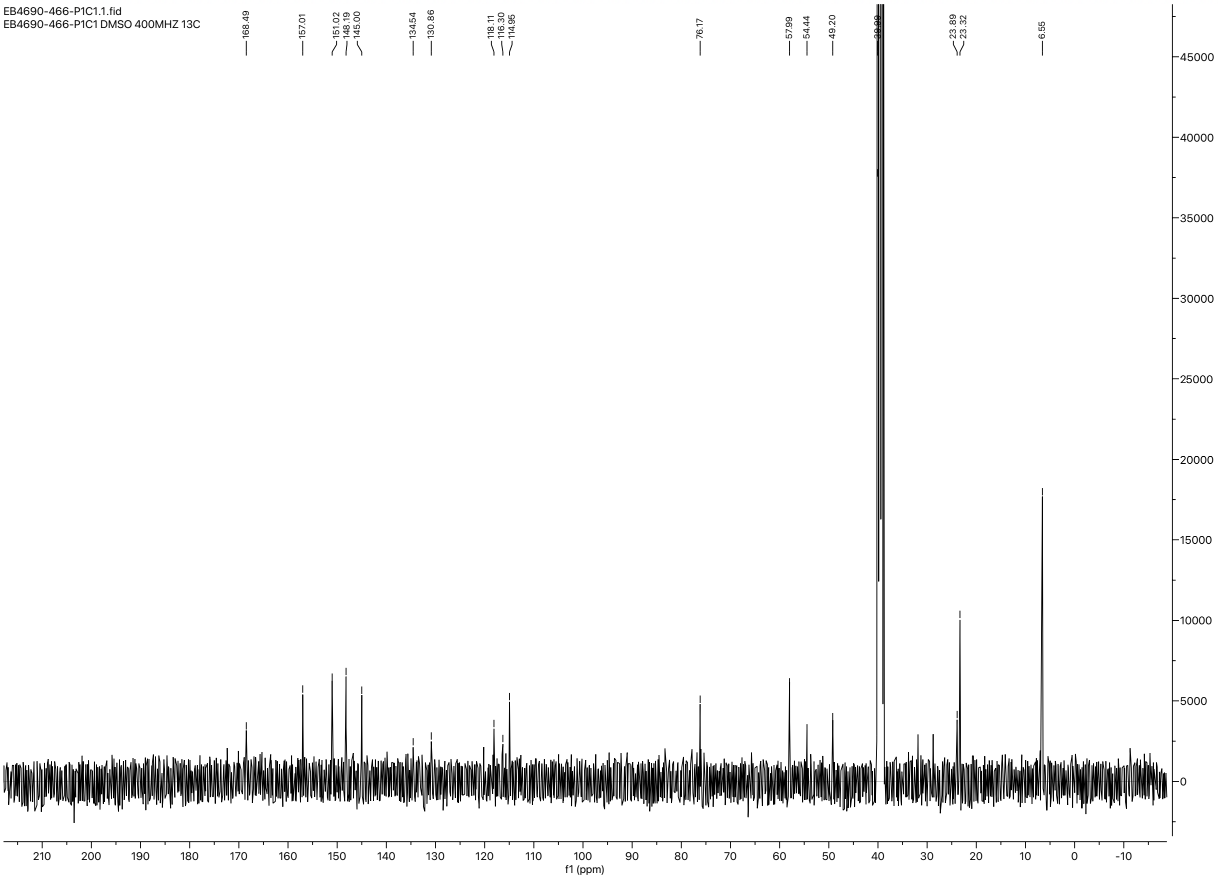

<Chromatogram>

Chromatogram
WX-TKDU-Y_027 D:\DATA\2022\2205\220512\EB4690-466-P1H1.lcd

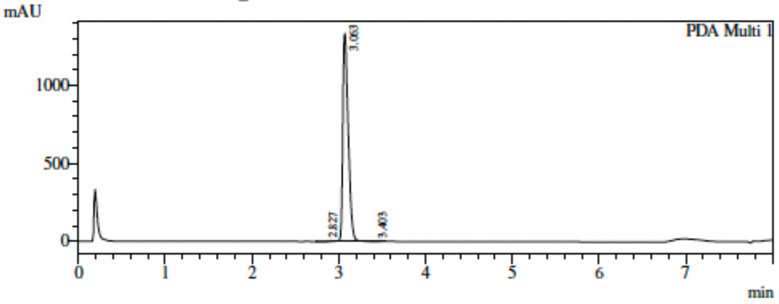

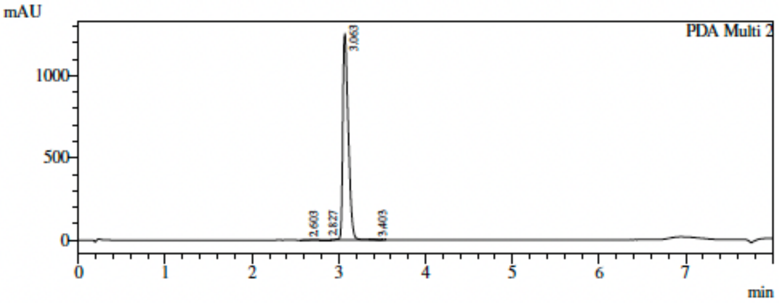

1 PDA Multi 1 / 220nm 4nm
2 PDA Multi 2 / 254nm 4nm

Integration Result

PeakTable

Ch1 220nm 4nm

Peak#	Ret. Time	Height	Height %	Area	Area %
1	2.827	537	0.040	3741	0.064
2	3.063	1335188	99.896	5817773	99.882
3	3.403	847	0.063	3116	0.054
Total		1336572	100.000	5824631	100.000

PeakTable

Ch2 254nm 4nm

Peak#	Ret. Time	Height	Height %	Area	Area %
1	2.603	311	0.025	2263	0.041
2	2.827	596	0.047	4191	0.076
3	3.063	1254815	99.850	5523486	99.812
4	3.403	981	0.078	3952	0.071
Total		1256703	100.000	5533892	100.000

PeakTable

Ch3

NMR and HPLC spectra of 2e:

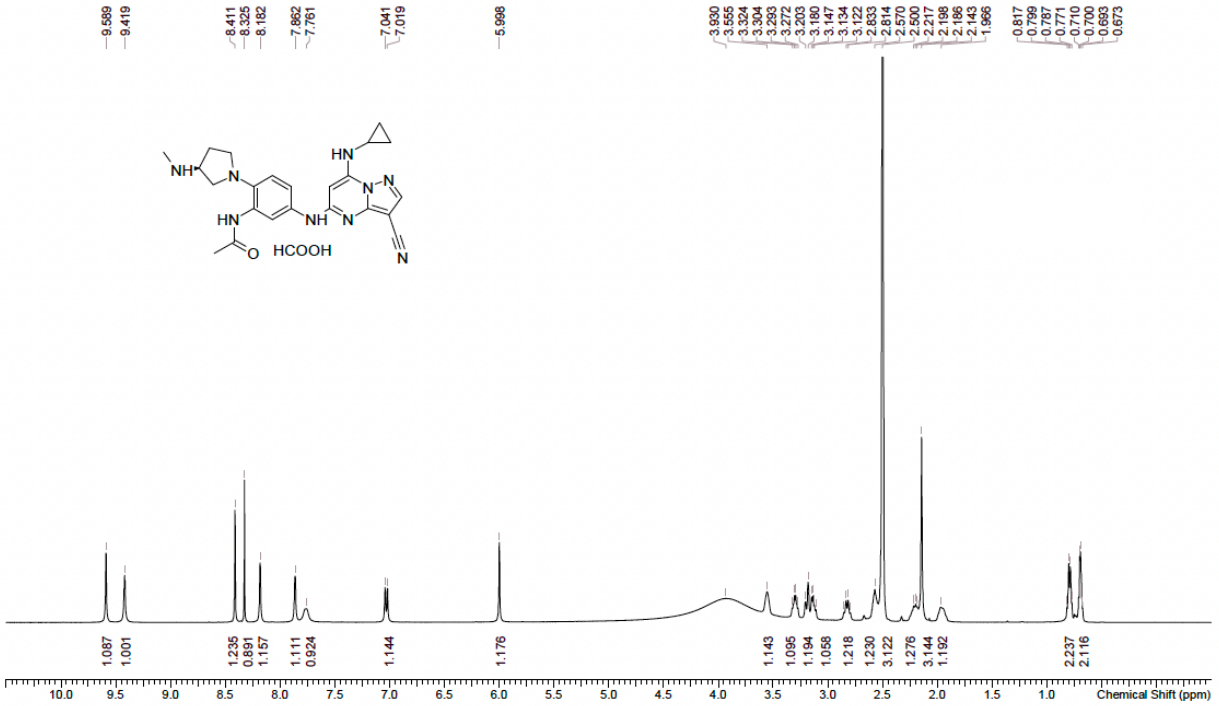

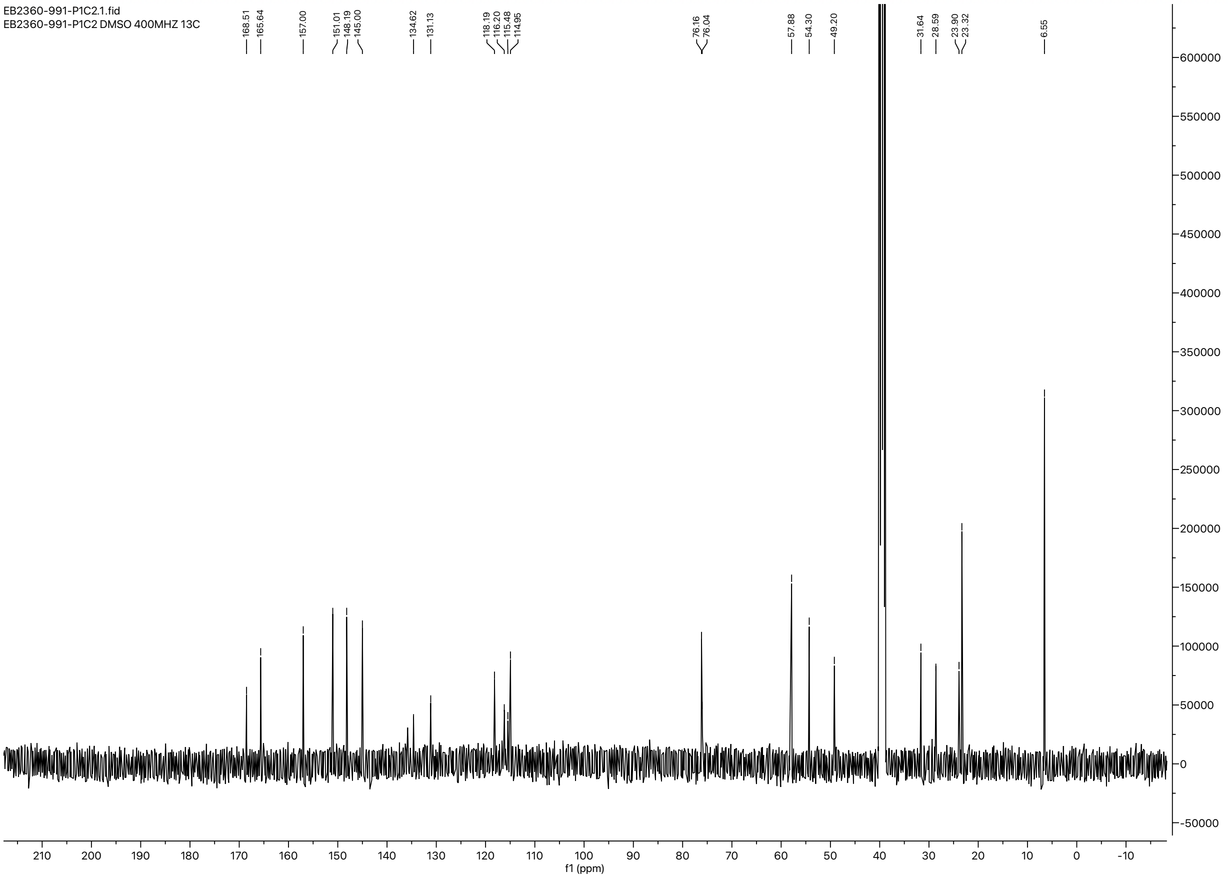

<Chromatogram>

Chromatogram
WX-TKDU-Y_028 D:\DATA\2022\2204\220429\EB2360-991-P1H2.lcd

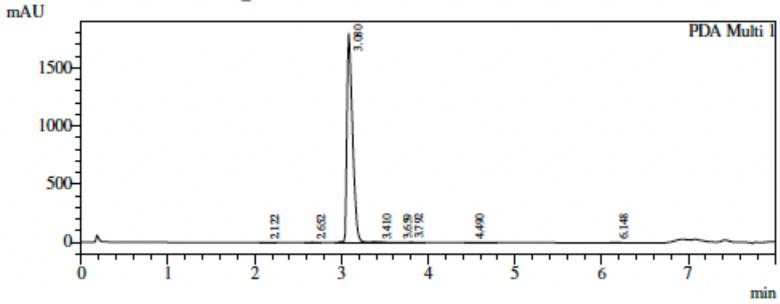

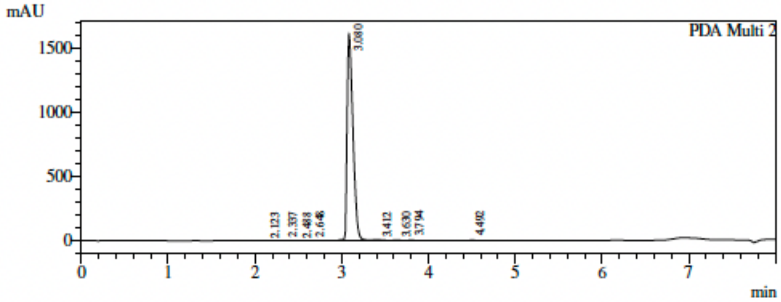

1 PDA Multi 1 / 220nm 4nm
2 PDA Multi 2 / 254nm 4nm

Integration Result

PeakTable

Ch1 220nm 4nm

Peak#	Ret. Time	Height	Height %	Area	Area %
1	2.122	879	0.049	3332	0.041
2	2.652	1488	0.082	6243	0.076
3	3.080	1795487	99.464	8126700	99.409
4	3.410	1132	0.063	4311	0.053
5	3.659	503	0.028	3100	0.038
6	3.792	1757	0.097	6196	0.076
7	4.490	1052	0.058	7485	0.092
8	6.148	2873	0.159	17664	0.216
Total		1805171	100.000	8175032	100.000

PeakTable

Ch2 254nm 4nm

Peak#	Ret. Time	Height	Height %	Area	Area %
1	2.123	367	0.023	1856	0.024
2	2.337	647	0.040	2470	0.032
3	2.488	678	0.042	2791	0.037
4	2.648	1147	0.071	5862	0.077
5	3.080	1618889	99.554	7573209	99.586
6	3.412	1503	0.092	5688	0.075
7	3.630	616	0.038	3590	0.047
8	3.794	1405	0.086	4974	0.065
9	4.492	886	0.054	4223	0.056
Total		1626137	100.000	7604665	100.000

PeakTable

Ch3

NMR and HPLC spectra of 2f:

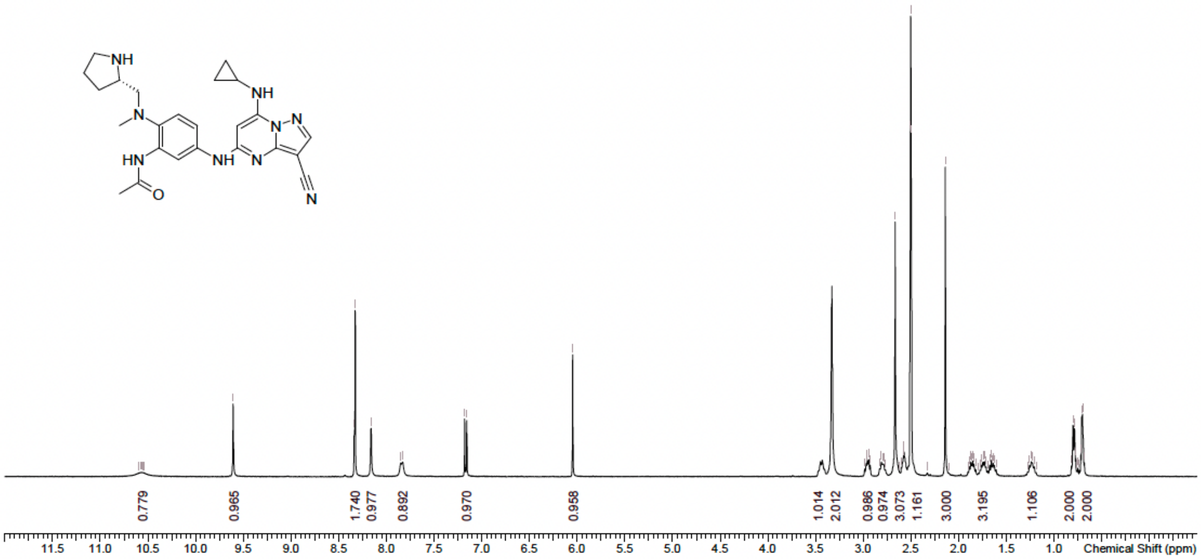

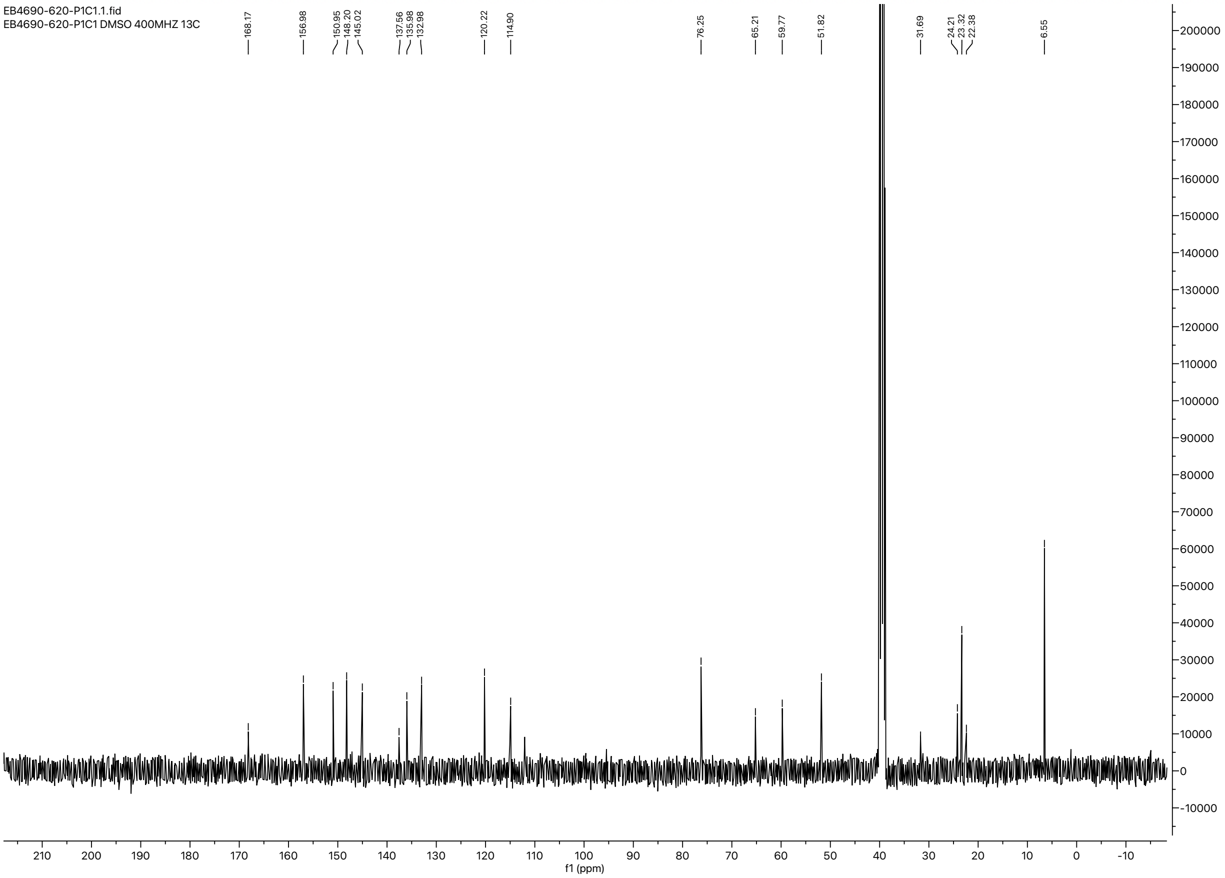

<Chromatogram>

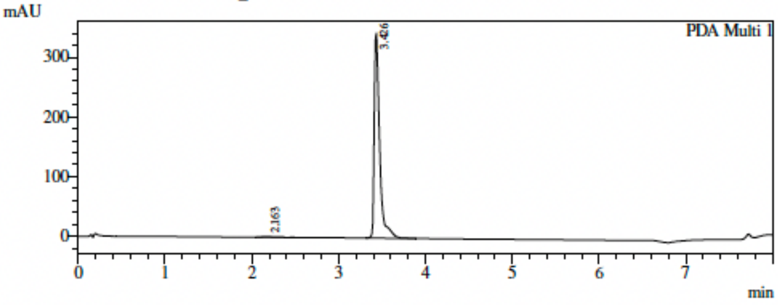

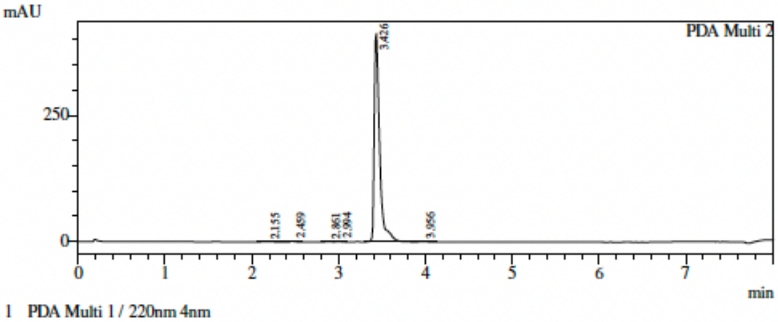

1 PDA Multi 1 / 220nm 4nm
2 PDA Multi 2 / 254nm 4nm

Integration Result

PeakTable

PDA Ch1 220nm 4nm

Peak#	Ret. Time	Height	Height %	Area	Area %
1	2.163	939	0.273	9684	0.645
2	3.426	343510	99.727	1491336	99.355
Total		344450	100.000	1501019	100.000

PeakTable

PDA Ch2 254nm 4nm

Peak#	Ret. Time	Height	Height %	Area	Area %
1	2.155	550	0.132	4596	0.254
2	2.459	1237	0.297	3627	0.200
3	2.861	741	0.178	2560	0.141
4	2.994	573	0.137	2121	0.117
5	3.426	412783	99.069	1792504	99.025
6	3.956	777	0.186	4750	0.262
Total		416661	100.000	1810158	100.000

PeakTable

PDA Ch3

NMR and HPLC spectra of 2g:

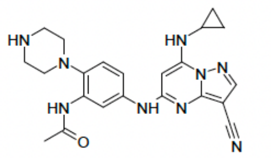

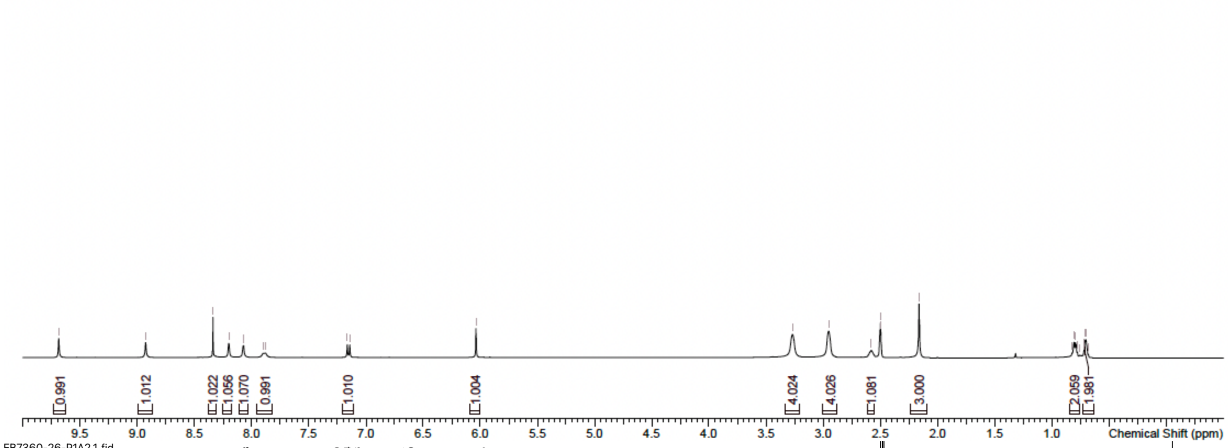

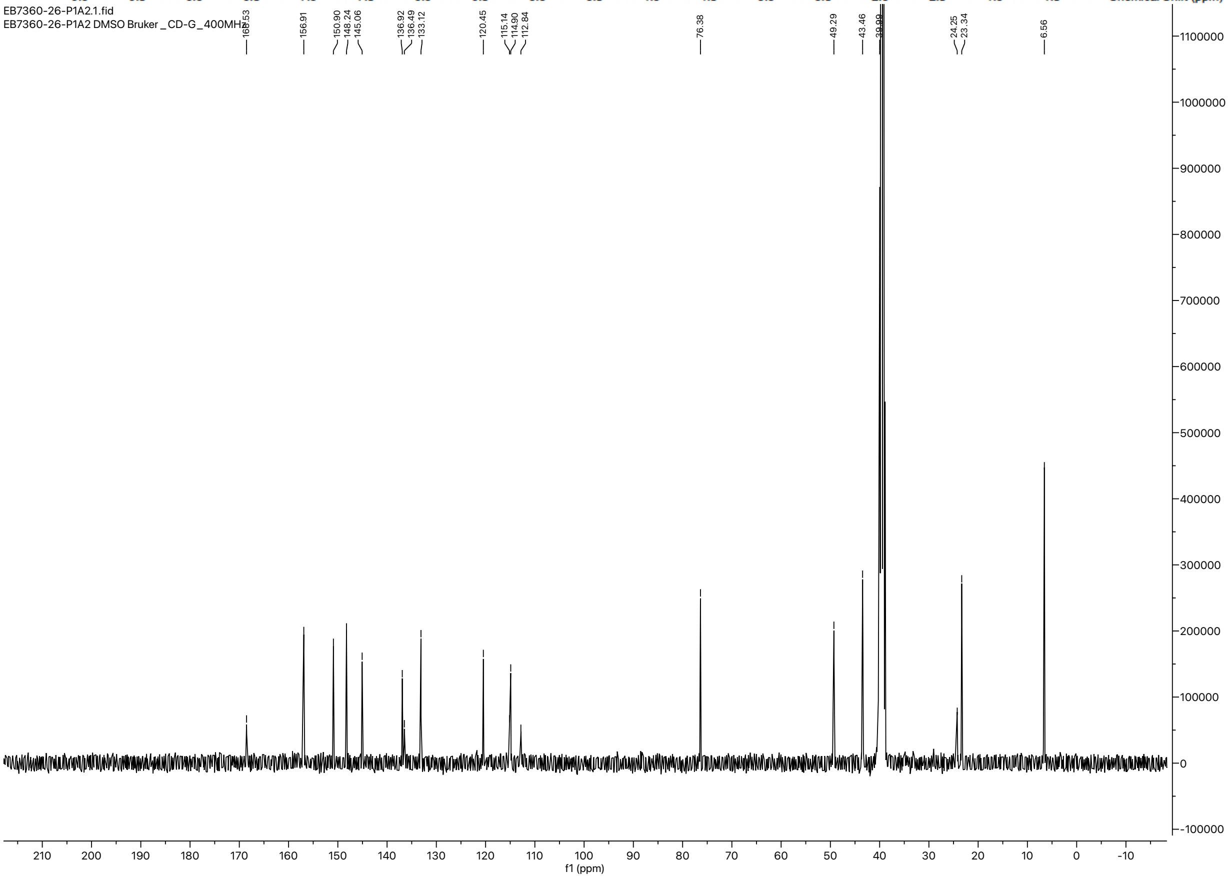

Chromatogram

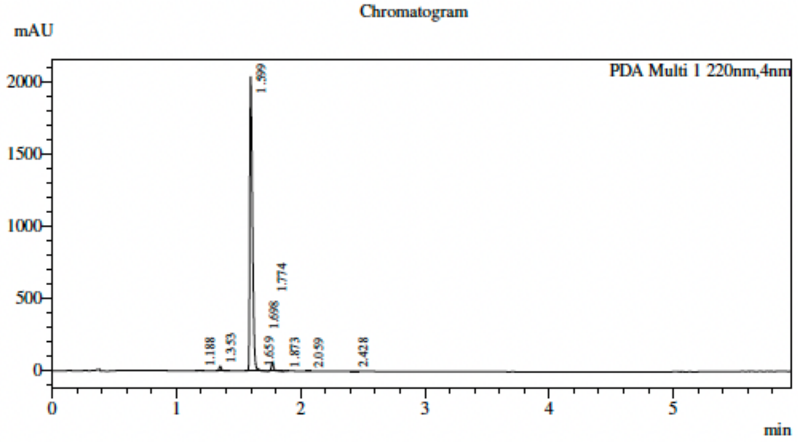

===

Integration Result

===

PDA Ch1 220nm

Peak#	Ret. Time	USP Width	Height	Height%	Area	Area%
1	1.188	0.029	2840	0.132	3255	0.102
2	1.353	0.031	31659	1.471	39398	1.233
3	1.599	0.040	2040631	94.802	3056550	95.644
4	1.659	0.346	10296	0.478	11494	0.360
5	1.698	0.154	2754	0.128	6020	0.188
6	1.774	0.031	59167	2.749	72616	2.272
7	1.873	0.033	2499	0.116	2954	0.092
8	2.059	0.030	1139	0.053	1324	0.041
9	2.428	0.037	1527	0.071	2137	0.067
Total			2152512	100.000	3195748	100.000

Chromatogram

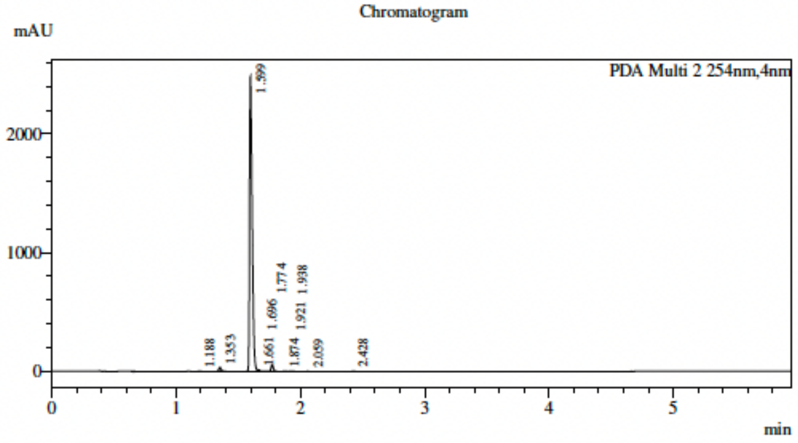

===

Integration Result

===

PDA Ch2 254nm

Peak#	Ret. Time	USP Width	Height	Height%	Area	Area%
1	1.188	0.029	2055	0.079	2348	0.061
2	1.353	0.031	33133	1.273	41448	1.082
3	1.599	0.040	2483078	95.403	3680072	96.064
4	1.661	0.132	13669	0.525	15936	0.416
5	1.696	0.103	3076	0.118	5824	0.152
6	1.774	0.031	57759	2.219	71882	1.876
7	1.874	0.038	2793	0.107	4013	0.105
8	1.921	0.071	2304	0.089	3326	0.087
9	1.938	0.178	2026	0.078	2223	0.058
10	2.059	0.031	1067	0.041	1231	0.032
11	2.428	0.038	1775	0.068	2539	0.066
Total			2602737	100.000	3830842	100.000

NMR and HPLC spectra of 2i:

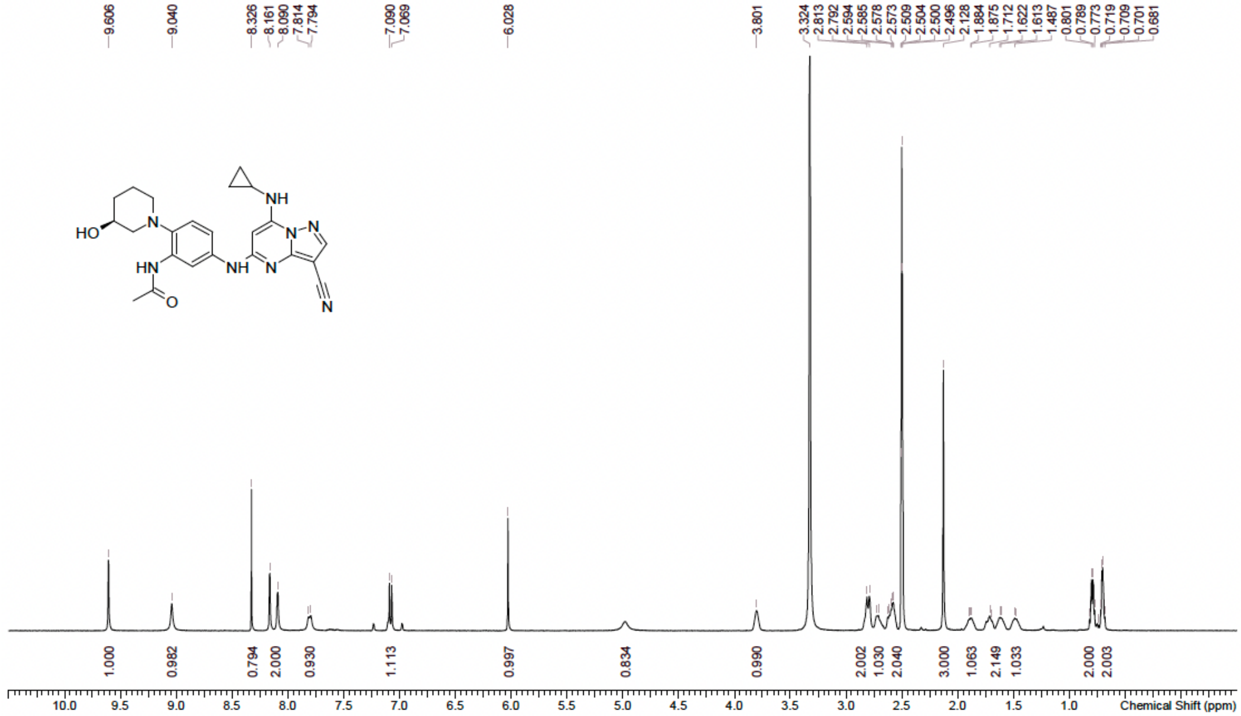

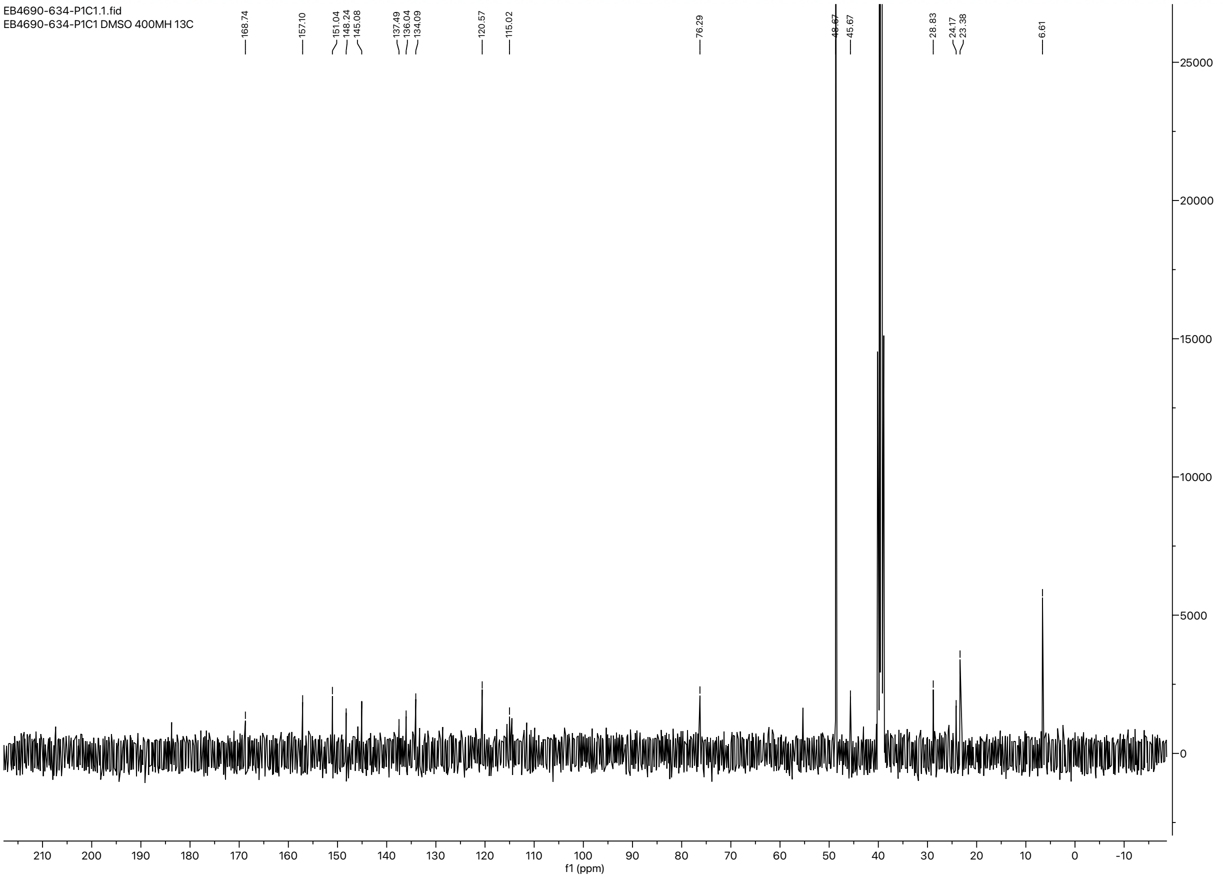

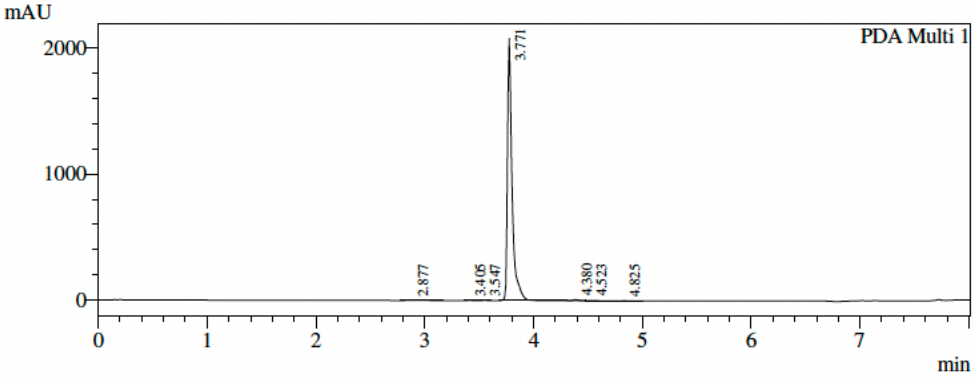

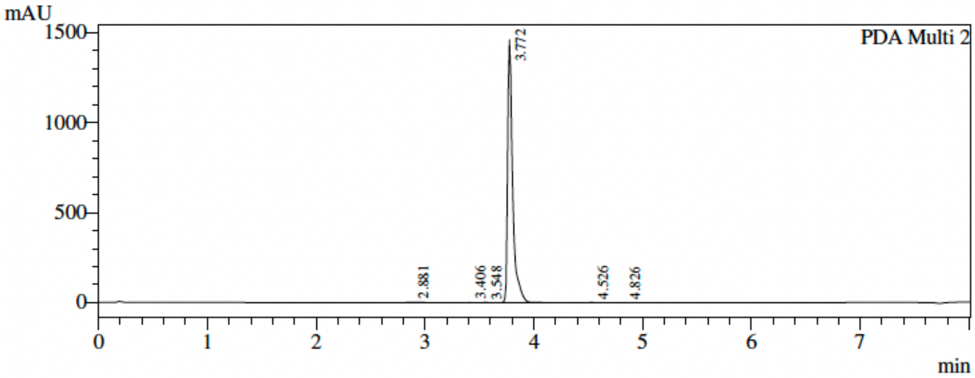

1 PDA Multi 1 / 220nm 4nm
2 PDA Multi 2 / 254nm 4nm

===
Integration Result
===

PeakTable

PDA Ch1 220nm 4nm

Peak#	Ret. Time	Height	Height %	Area	Area %
1	2.877	1163	0.056	12629	0.206
2	3.405	1255	0.060	3405	0.056
3	3.547	1153	0.055	3183	0.052
4	3.771	2077942	99.328	6071865	98.995
5	4.380	7986	0.382	30941	0.504
6	4.523	1775	0.085	8301	0.135
7	4.825	725	0.035	3158	0.051
Total		2091998	100.000	6133482	100.000

PeakTable

PDA Ch2 254nm 4nm

Peak#	Ret. Time	Height	Height %	Area	Area %
1	2.881	1347	0.092	15542	0.330
2	3.406	768	0.052	2115	0.045
3	3.548	1061	0.072	2893	0.061
4	3.772	1459960	99.699	4685327	99.465
5	4.526	647	0.044	2310	0.049
6	4.826	584	0.040	2325	0.049
Total		1464367	100.000	4710512	100.000

PeakTable

PDA Ch3

NMR and HPLC spectra of 2j:

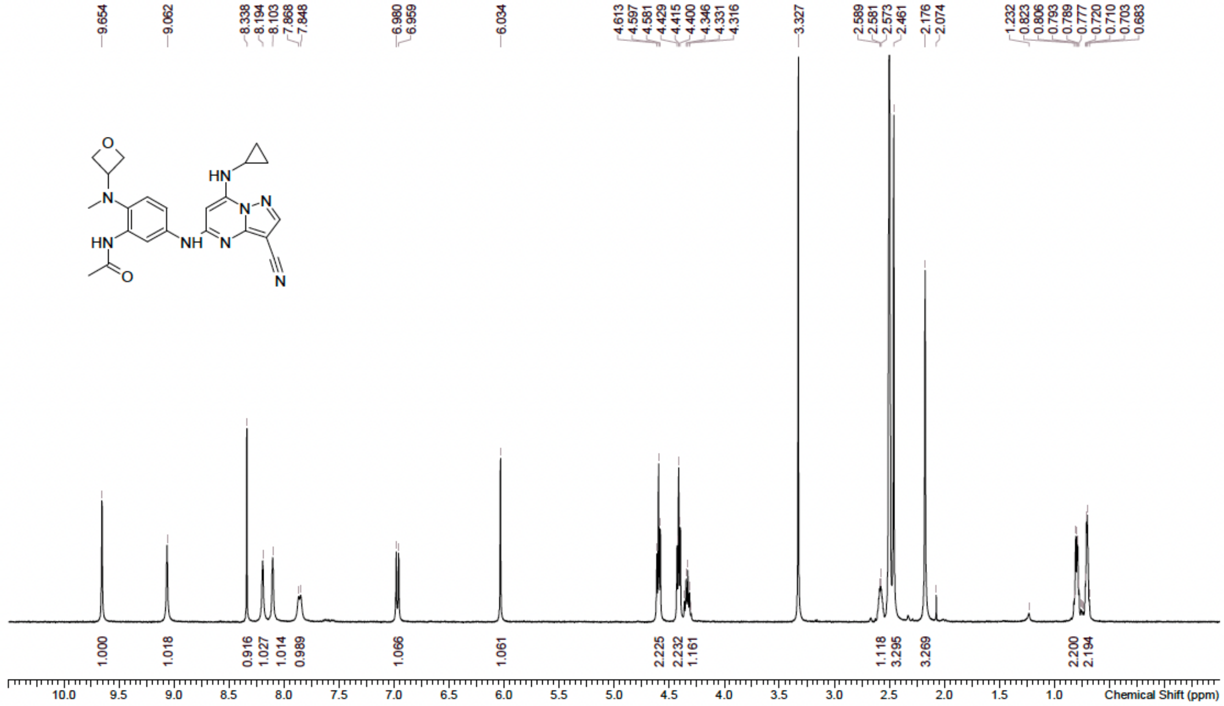

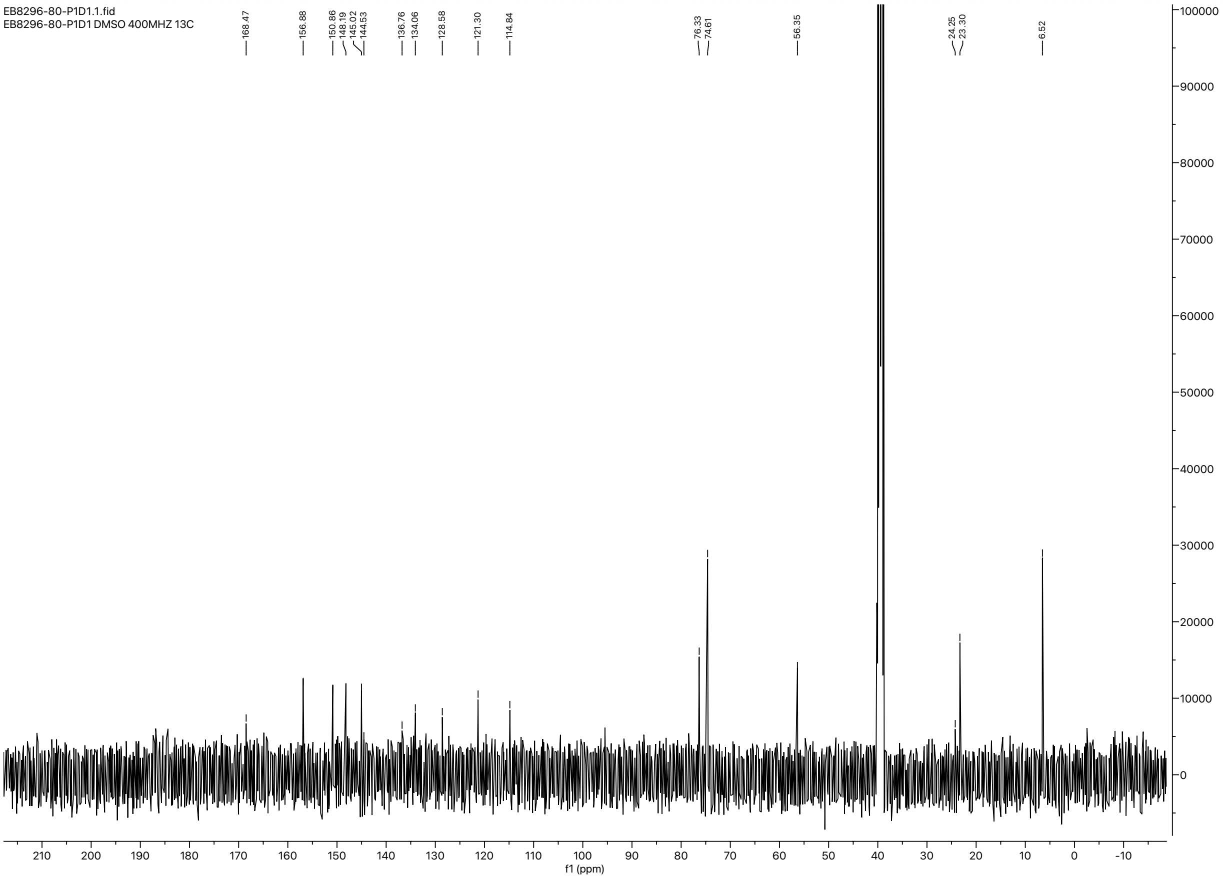

[illegible]

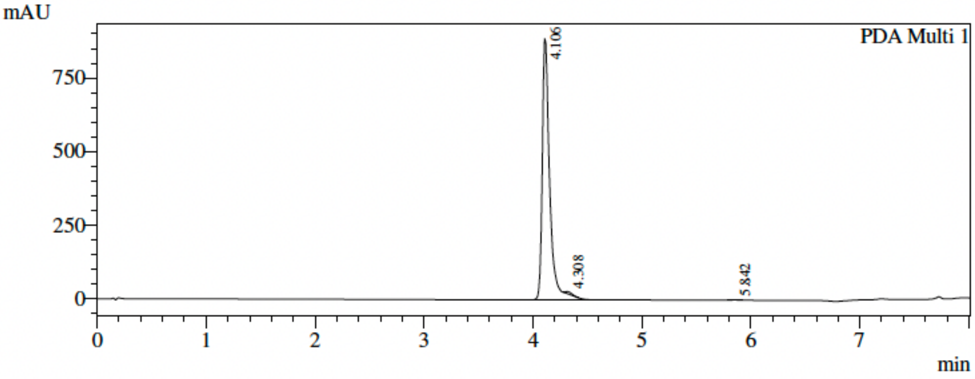

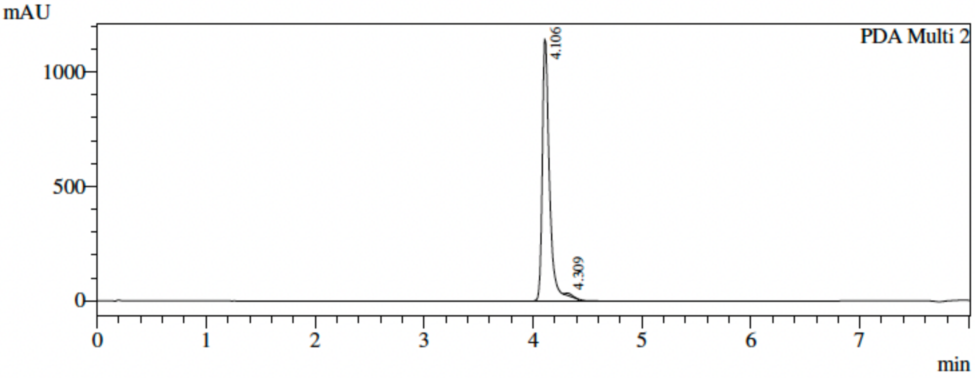

1 PDA Multi 1 / 220nm 4nm
2 PDA Multi 2 / 254nm 4nm

Integration Result

PeakTable

PDA Ch1 220nm 4nm

Peak#	Ret. Time	Height	Height %	Area	Area %
1	4.106	886833	99.264	4203036	99.335
2	4.308	5995	0.671	25736	0.608
3	5.842	583	0.065	2384	0.056
Total		893411	100.000	4231157	100.000

PeakTable

PDA Ch2 254nm 4nm

Peak#	Ret. Time	Height	Height %	Area	Area %
1	4.106	1143554	99.264	5337382	99.318
2	4.309	8480	0.736	36639	0.682
Total		1152034	100.000	5374021	100.000

PeakTable

PDA Ch3

NMR and HPLC spectra of 2k:

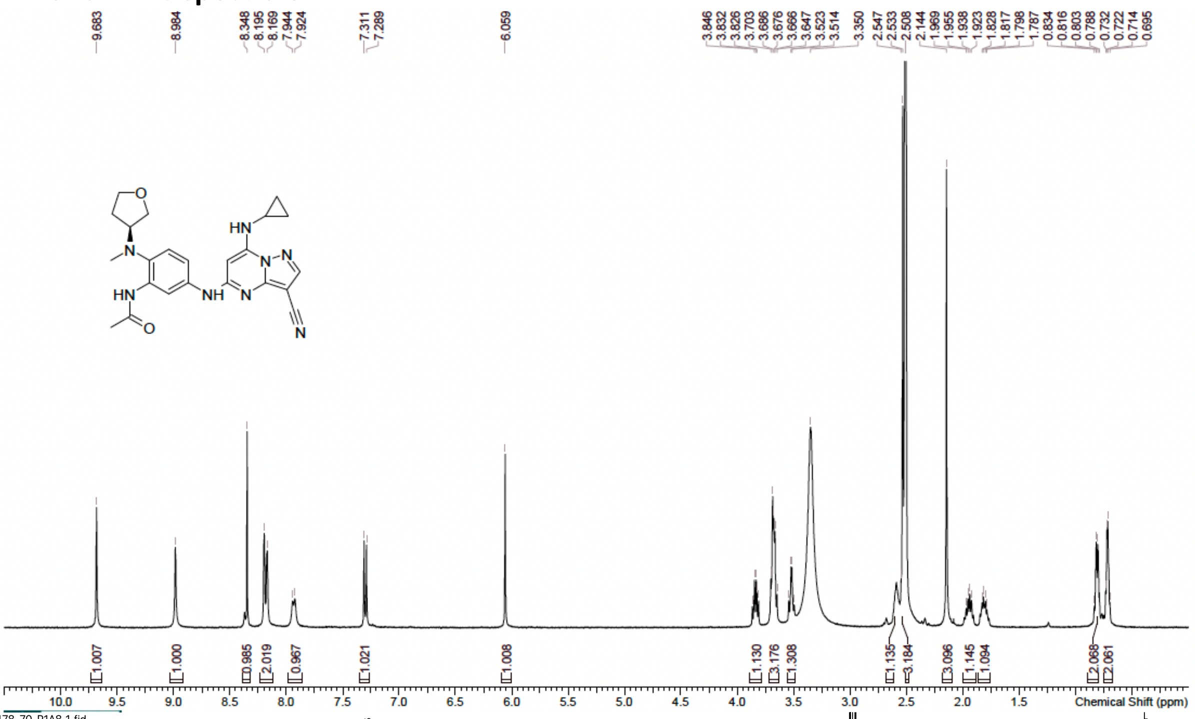

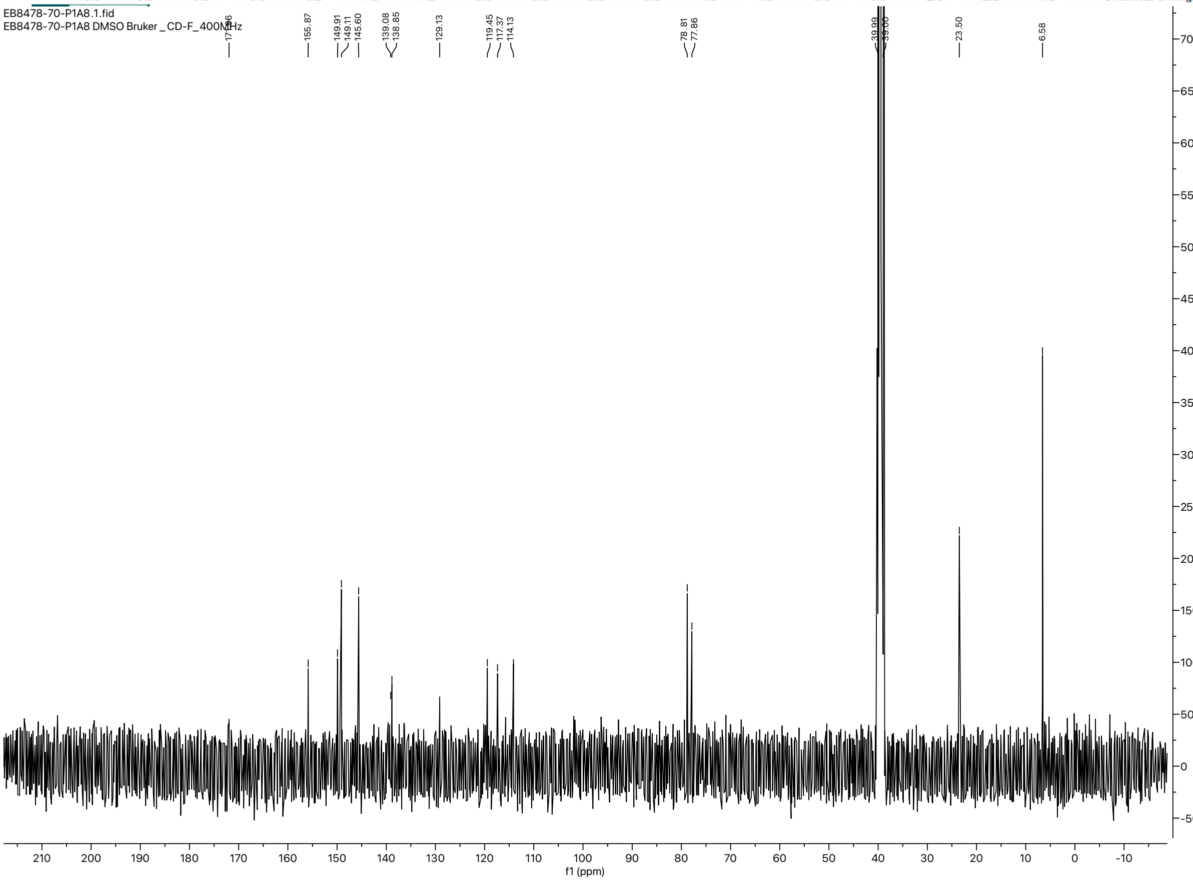

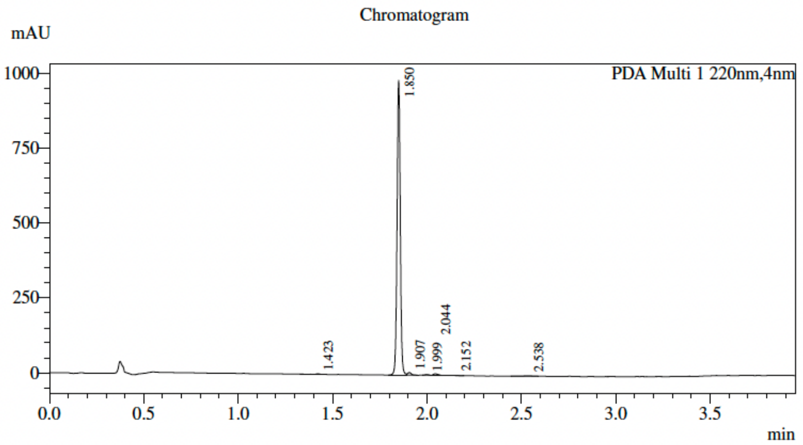

Integration Result

PDA Ch1 220nm

Peak#	Ret. Time	USP Width	Height	Height%	Area	Area%
1	1.423	0.027	1466	0.146	1475	0.122
2	1.850	0.031	985161	97.960	1174514	97.374
3	1.907	0.037	8679	0.863	11739	0.973
4	1.999	0.032	2189	0.218	2584	0.214
5	2.044	0.043	5527	0.550	9142	0.758
6	2.152	0.109	1245	0.124	1800	0.149
7	2.538	0.105	1407	0.140	4939	0.409
Total			1005673	100.000	1206194	100.000

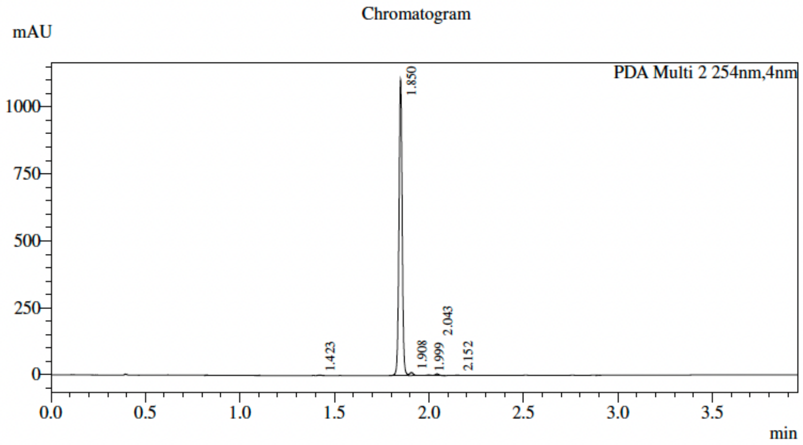

Integration Result

PDA Ch2 254nm

Peak#	Ret. Time	USP Width	Height	Height%	Area	Area%
1	1.423	0.028	2106	0.187	2149	0.160
2	1.850	0.031	1106064	98.001	1316884	97.824
3	1.908	0.036	11203	0.993	15210	1.130
4	1.999	0.033	2199	0.195	2634	0.196
5	2.043	0.032	5955	0.528	7441	0.553
6	2.152	0.040	1102	0.098	1854	0.138
Total			1128629	100.000	1346173	100.000

NMR and HPLC spectra of 2l:

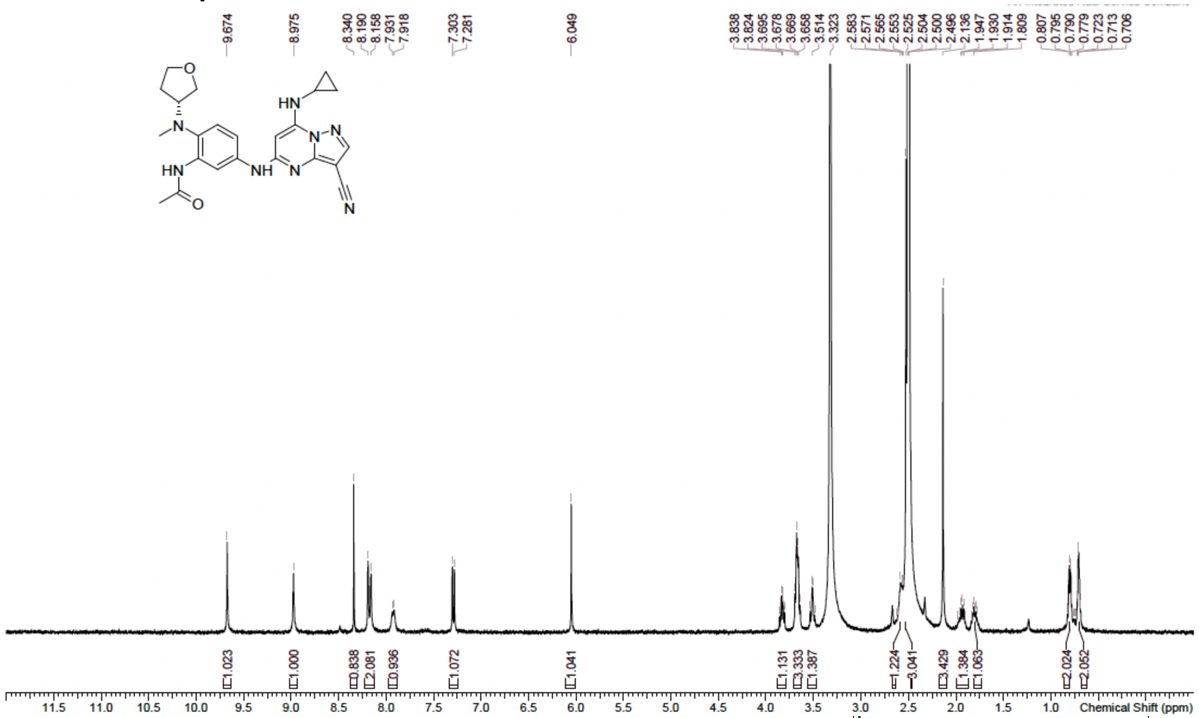

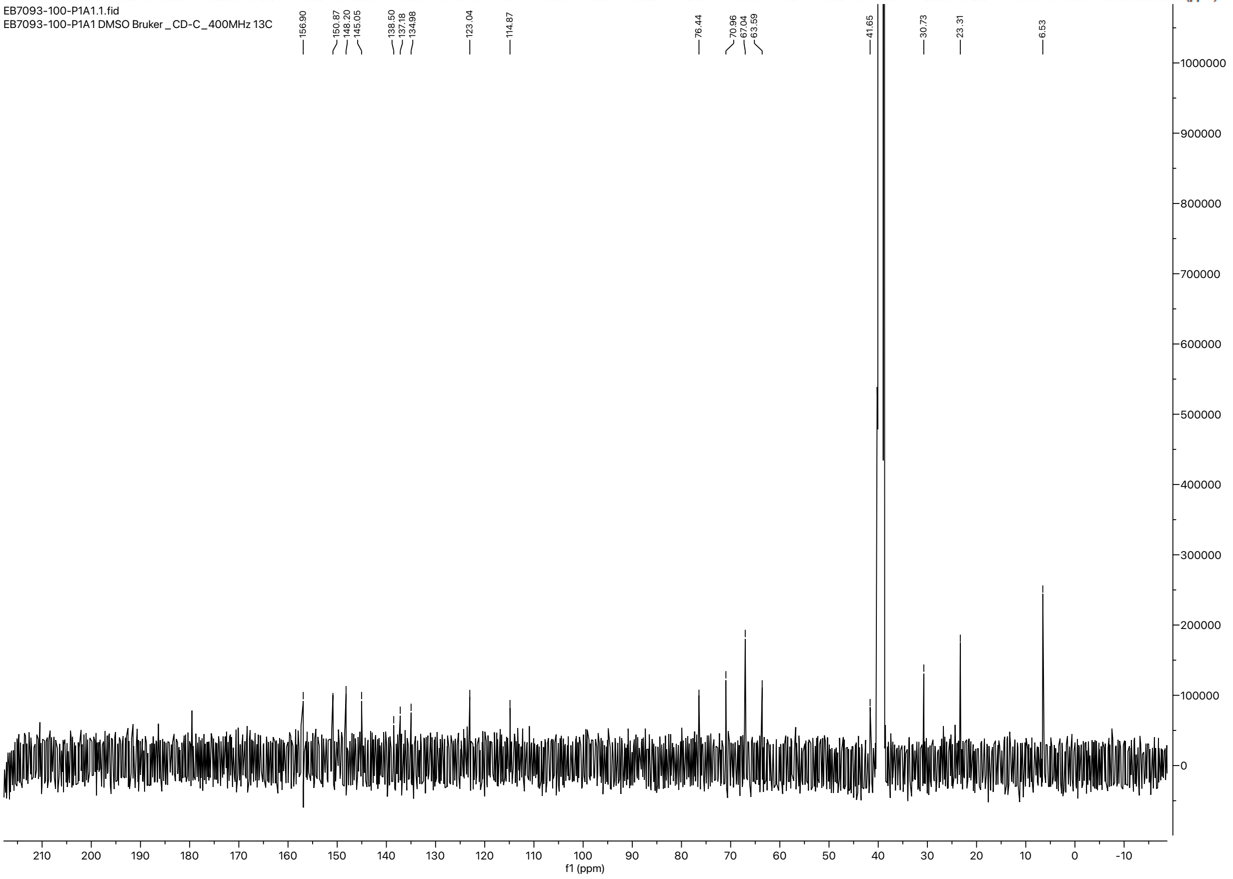

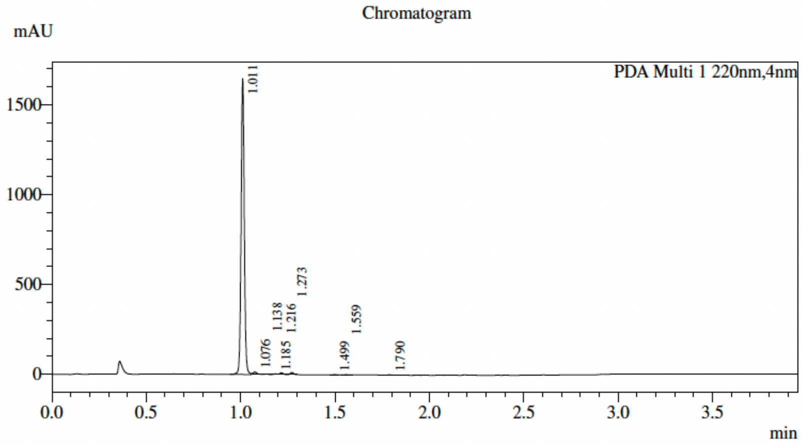

Integration Result

PDA Ch1 220nm

Peak#	Ret. Time	USP Width	Height	Height%	Area	Area%
1	1.011	0.030	1647850	97.064	1930550	96.685
2	1.076	0.040	13718	0.808	21639	1.084
3	1.138	0.035	2810	0.166	3740	0.187
4	1.185	0.036	4091	0.241	5236	0.262
5	1.216	0.034	10313	0.607	13133	0.658
6	1.273	0.033	11686	0.688	14086	0.705
7	1.499	0.032	2509	0.148	3055	0.153
8	1.559	0.032	2420	0.143	3133	0.157
9	1.790	0.025	2291	0.135	2180	0.109
Total			1697688	100.000	1996752	100.000

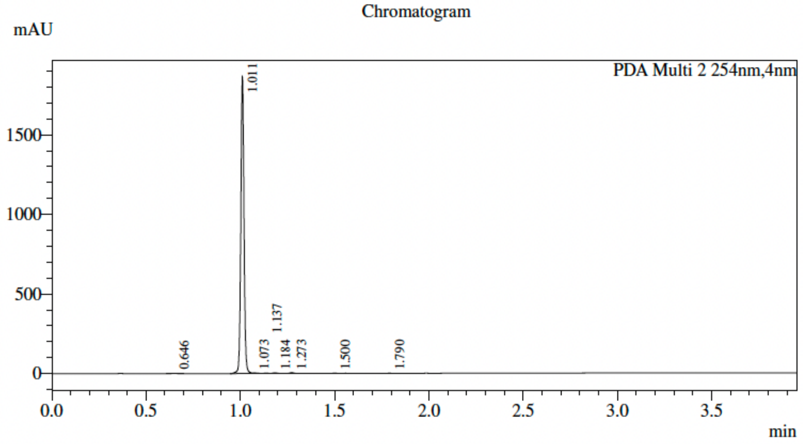

Integration Result

PDA Ch2 254nm

Peak#	Ret. Time	USP Width	Height	Height%	Area	Area%
1	0.646	0.040	1654	0.088	2754	0.125
2	1.011	0.030	1863169	98.883	2179383	98.756
3	1.073	0.061	4213	0.224	6923	0.314
4	1.137	0.030	2226	0.118	2587	0.117
5	1.184	0.032	4371	0.232	5208	0.236
6	1.273	0.033	5366	0.285	6454	0.292
7	1.500	0.031	2156	0.114	2586	0.117
8	1.790	0.024	1053	0.056	943	0.043
Total			1884208	100.000	2206839	100.000

NMR and HPLC spectra of 2m:

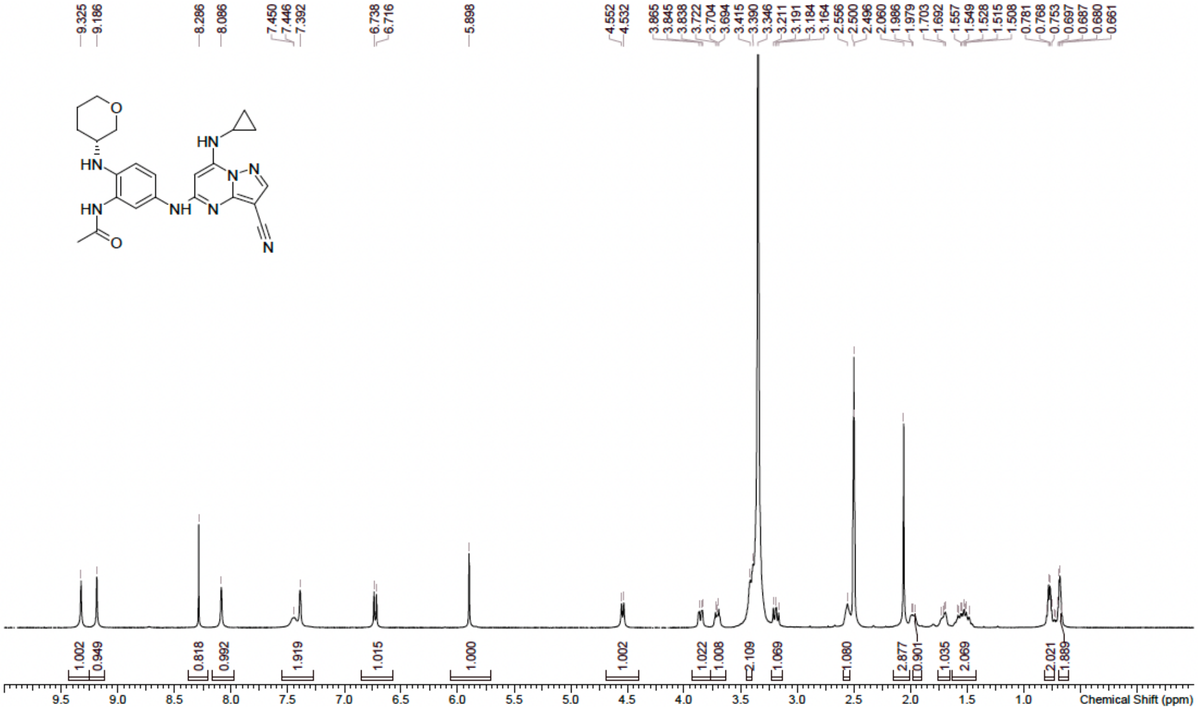

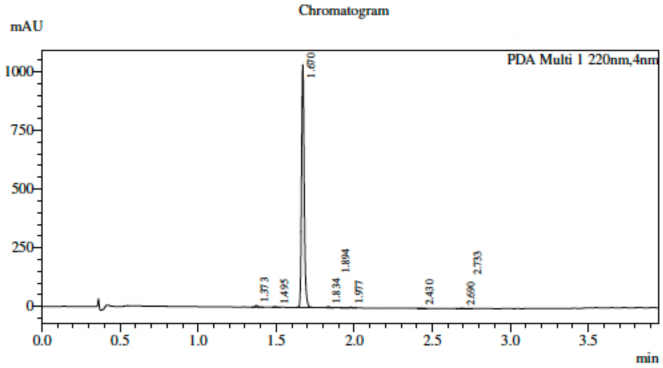

Integration Result

PDA Ch1 220nm

Peak#	Ret. Time	USP Width	Height	Height%	Area	Area%
1	1.373	0.028	7323	0.693	8110	0.653
2	1.495	0.031	2975	0.281	3892	0.314
3	1.670	0.030	1034702	97.890	1211514	97.599
4	1.834	0.032	4392	0.416	5782	0.466
5	1.894	0.041	1102	0.104	1807	0.146
6	1.977	0.034	2849	0.270	4428	0.357
7	2.430	0.042	1239	0.117	1969	0.159
8	2.690	0.037	1362	0.129	2147	0.173
9	2.733	0.041	1055	0.100	1674	0.135
Total			1057000	100.000	1241322	100.000

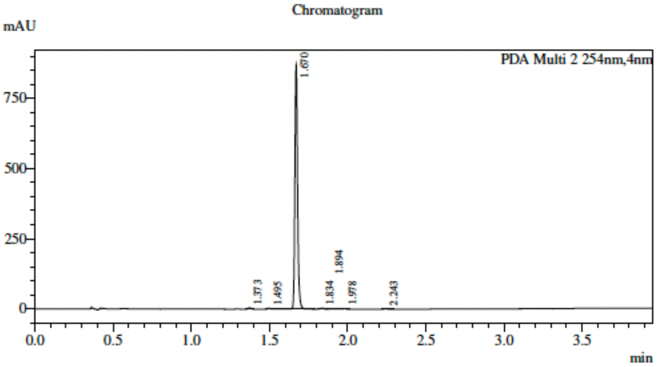

Integration Result

PDA Ch2 254nm

Peak#	Ret. Time	USP Width	Height	Height%	Area	Area%
1	1.373	0.028	4165	0.470	4449	0.427
2	1.495	0.031	2984	0.337	5124	0.492
3	1.670	0.030	871955	98.352	1021765	98.038
4	1.834	0.031	3351	0.378	4028	0.386
5	1.894	0.042	1278	0.144	2881	0.276
6	1.978	0.035	1812	0.204	2479	0.238
7	2.243	0.037	1024	0.116	1488	0.143
Total			886569	100.000	1042213	100.000

NMR and HPLC spectra of 2n:

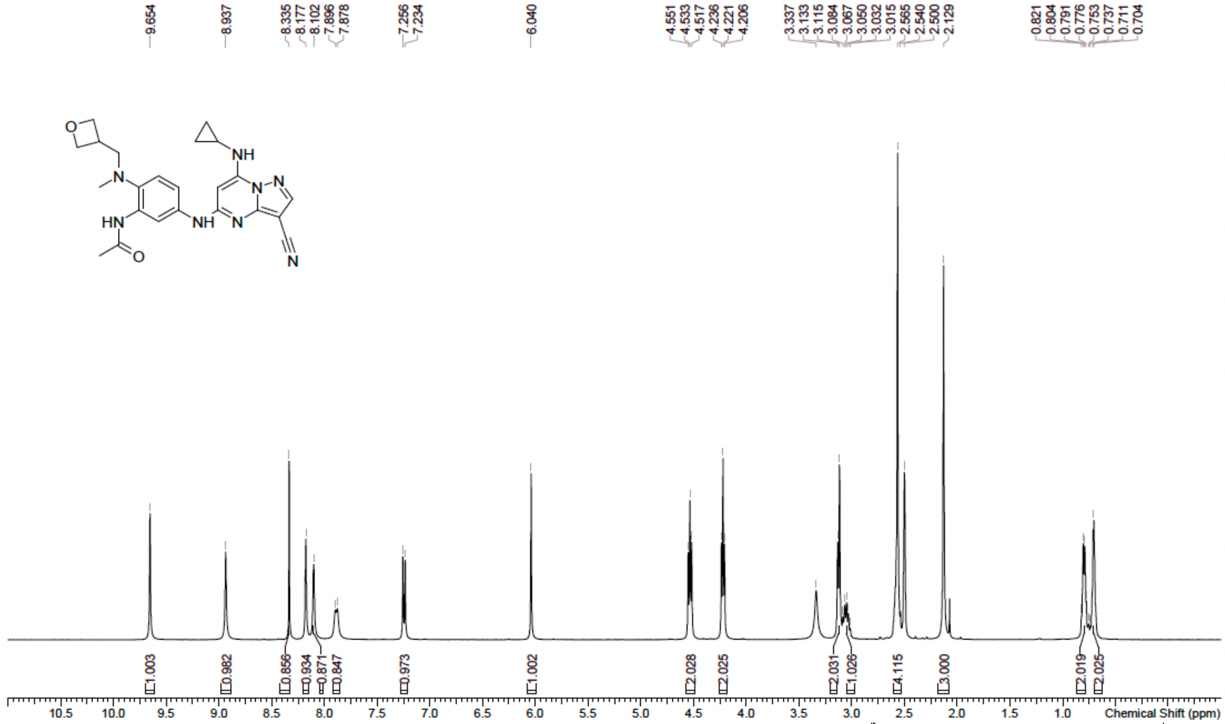

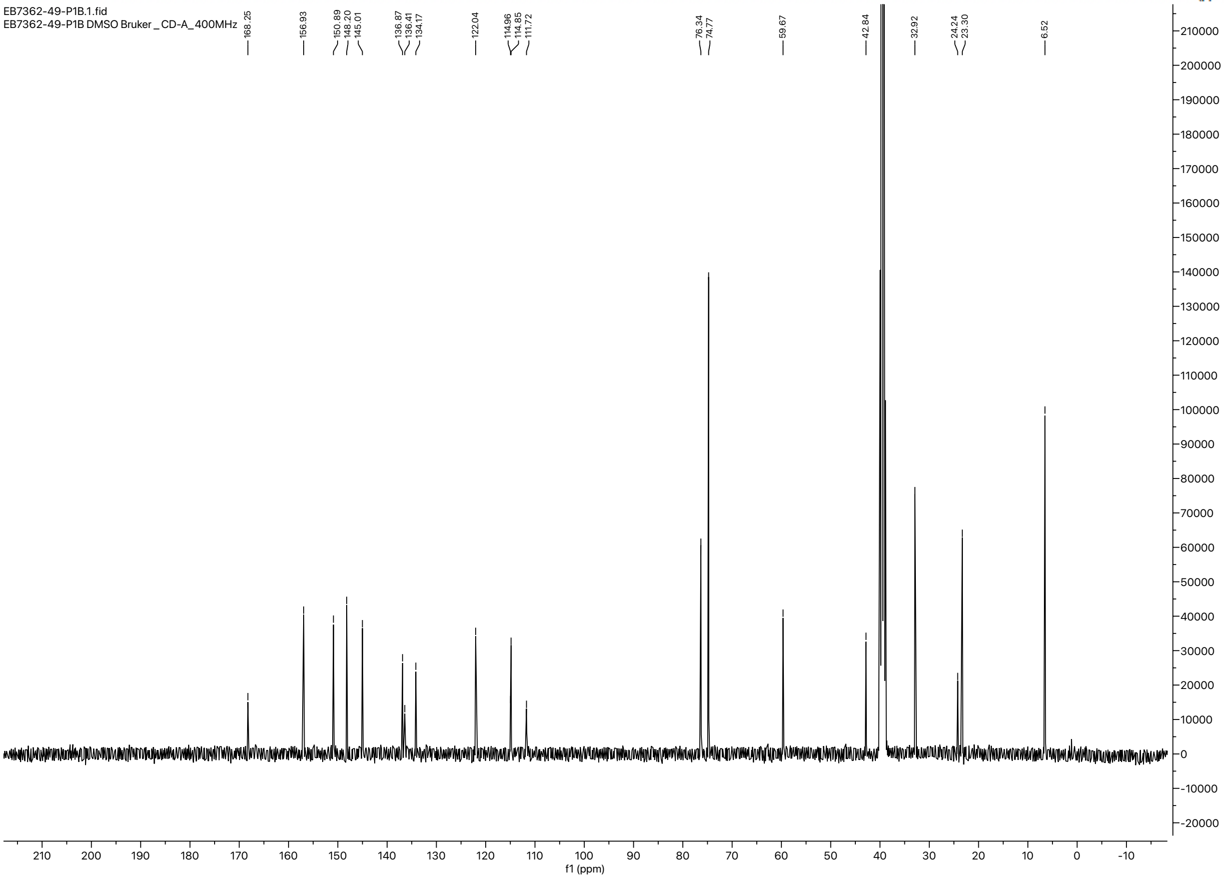

Chromatogram

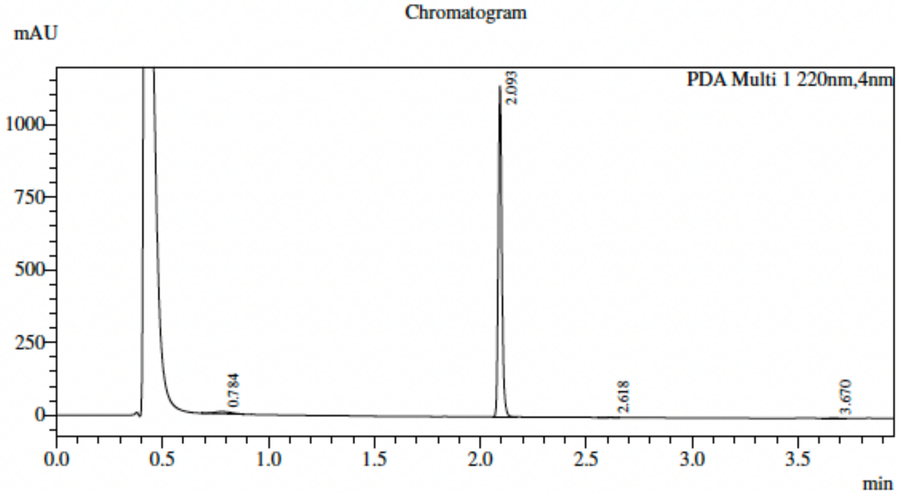

Integration Result

PDA Ch1 220nm

Peak#	Ret. Time	USP Width	Height	Height%	Area	Area%
1	0.784	0.136	7387	0.643	40781	2.928
2	2.093	0.030	1139441	99.175	1346604	96.681
3	2.618	0.051	1021	0.089	2485	0.178
4	3.670	0.059	1071	0.093	2960	0.213
Total			1148920	100.000	1392830	100.000

Chromatogram

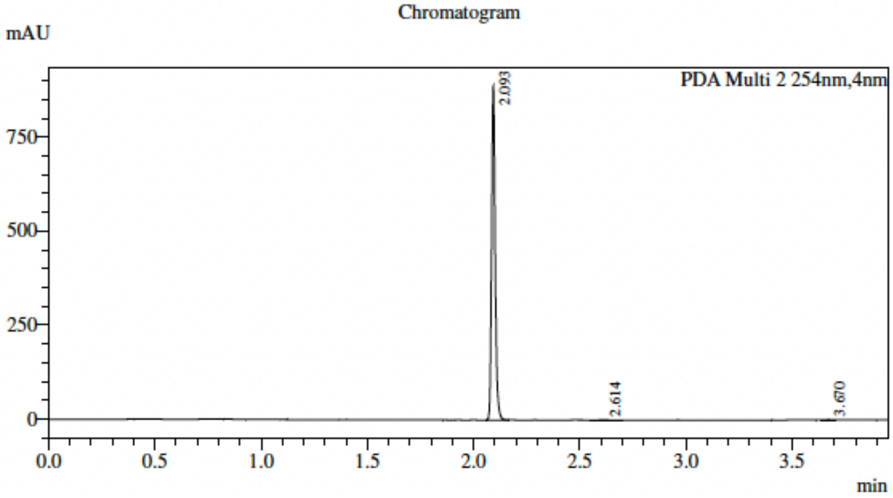

Integration Result

PDA Ch2 254nm

Peak#	Ret. Time	USP Width	Height	Height%	Area	Area%
1	2.093	0.030	887654	99.743	1051968	99.517
2	2.614	0.051	1138	0.128	3058	0.289
3	3.670	0.044	1148	0.129	2043	0.193
Total			889941	100.000	1057069	100.000
